# Supplementary material for: Bayesian nonparametric discovery of isoforms and individual specific quantification
Source: Nat Commun. 2018 Apr 27;9:1681. doi: 10.1038/s41467-018-03402-w (PMC5923247; doi:10.1038/s41467-018-03402-w)
Supplement: Supplementary file 1 — Supplementary Information [file 41467_2018_3402_MOESM1_ESM.pdf]

Bayesian nonparametric discovery of isoforms and individual specific quantification

Aguiar *et al.*

Supplementary Figure 1	An example to demonstrate isoform discovery evaluation criteria.
Supplementary Figure 2	Average coverage per gene density plot.
Supplementary Figure 3	Isoform discovery precision and recall for central exons in BEERS simulated data.
Supplementary Figure 4	Recall and precision for BEERS data by number of transcripts.
Supplementary Figure 5	Recall and precision for BEERS data by number of exons.
Supplementary Figure 6	The number of correctly inferred and expressed transcripts in BEERS data by exon composition.
Supplementary Figure 7	The number of correctly inferred and expressed transcripts in BEERS data by coverage.
Supplementary Figure 8	Isoform quantification accuracy on high coverage isoforms.
Supplementary Figure 9	Read aligner performance (precision) on Iso-Seq data.
Supplementary Figure 10	Read aligner performance (true positives) on Iso-Seq data.
Supplementary Figure 11	Correlation of expression values for shared transcripts across methods.
Supplementary Figure 12	The number of correctly inferred and expressed transcripts in Iso-Seq data by exon composition.
Supplementary Figure 13	The number of correctly inferred and expressed transcripts in Iso-Seq data by span.
Supplementary Figure 14	Method runtimes as a function of the number of exons on Iso-Seq data.
Supplementary Figure 15	Method runtimes as a function of gene length on Iso-Seq data.
Supplementary Figure 16	Method runtimes as a function of read length on Iso-Seq data.
Supplementary Figure 17	Method runtimes as a function of span on Iso-Seq data.

Supplementary Figure 18	The density of genes with varying numbers of alternative isoforms by (left) population and (right) sex.
Supplementary Figure 19	Histogram of BIISQ runtimes and memory usage for GEUVADIS data.
Supplementary Figure 20	A graphical model for the generation of reads in RNA-seq data from M samples.
Supplementary Figure 21	Genotype PCs for Geuvadis data.
Supplementary Figure 22	Theoretical vs. sample quantiles for variant enrichment.
Supplementary Table 1	Exon coverage for BEERS data.
Supplementary Table 2	Iso-Seq gold standard.
Supplementary Table 3	Variant enrichment sample descriptions.
Supplementary Table 4	Functionary categories for enrichment analysis.
Supplementary Table 5	Functional enrichment for genes with at least one trQTL.
Supplementary Table 6	Functional enrichment for isoforms with > 1 transcripts.
Supplementary Table 7	Functional enrichment for isoforms with > 4 transcripts.
Supplementary Table 8	Functional enrichment for isoforms with > 6 transcripts.
Supplementary Table 9	Model and variational parameters, conditional distributions and expectations.
Supplementary Table 10	Updates for variational parameters in the BIISQ model.
Supplementary Table 11	PEER factor optimization.
Supplementary Note	Read mapping likelihood
Supplementary Methods	Additional details on the BIISQ model, posterior inference, and simulations.

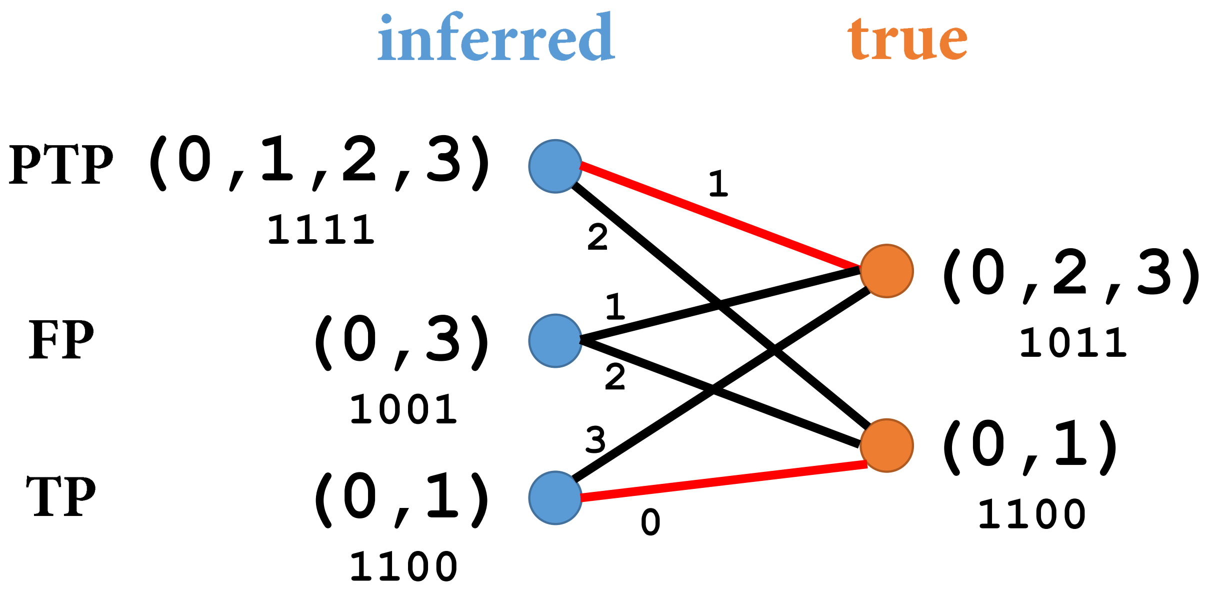

Supplementary Figure 1: **An example to demonstrate isoform discovery evaluation criteria.** Algorithmically inferred isoforms are shown on the left; true isoforms are depicted on the right. The maximum cardinality minimum weight matching between the inferred and true isoforms is shown in orange edges and defines pairs of inferred and true isoforms that are maximally similar. Pairs with exon distance > 0 are partial true positives (PTP), with distance $= 0$ are true positives, and inferred isoforms without a matching true isoform are false positives.

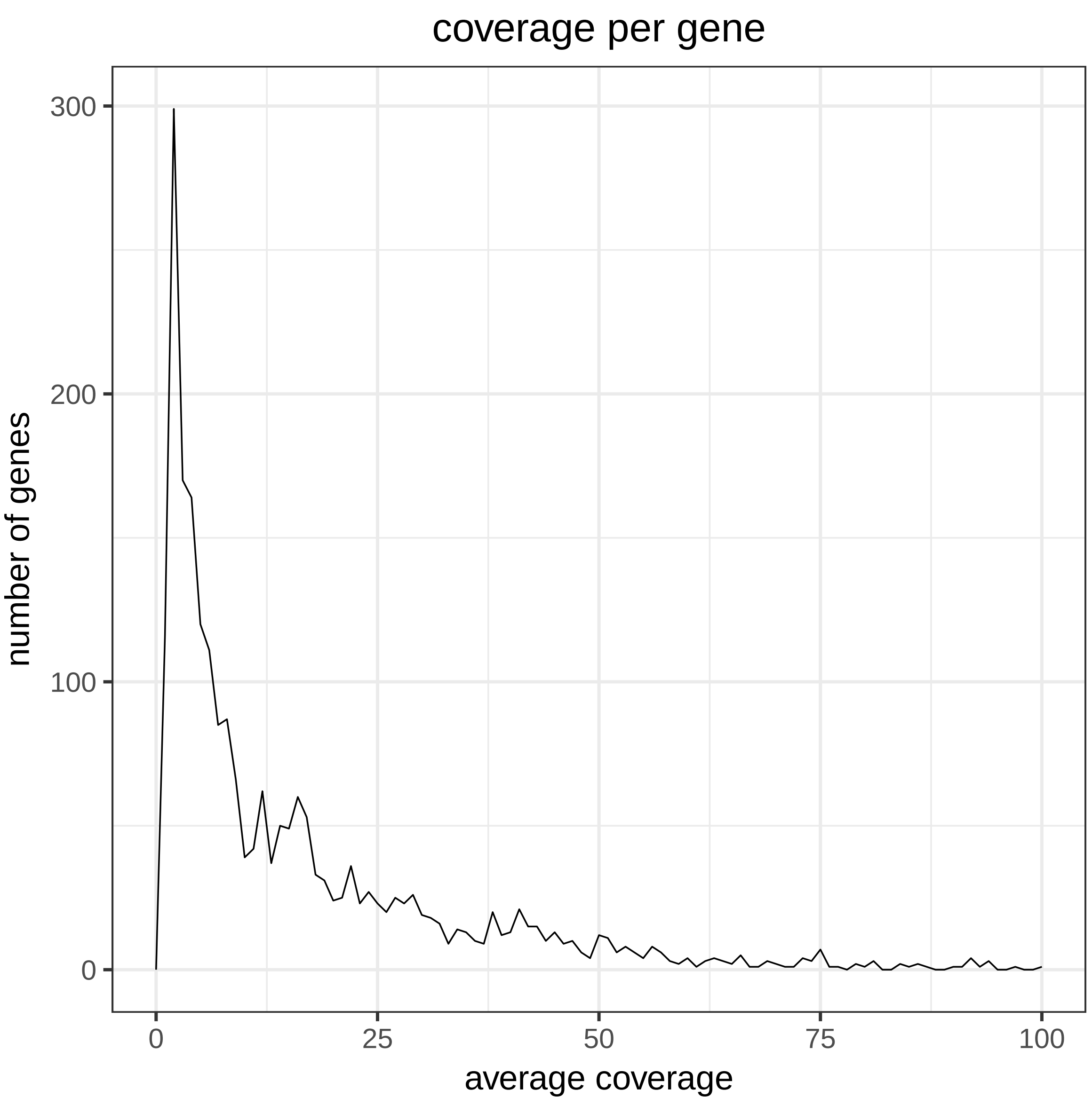

Supplementary Figure 2: **Average coverage per gene density plot.** Density (in terms of gene count) plot of gene coverage averaged across individuals and isoforms in BEERS simulated data.

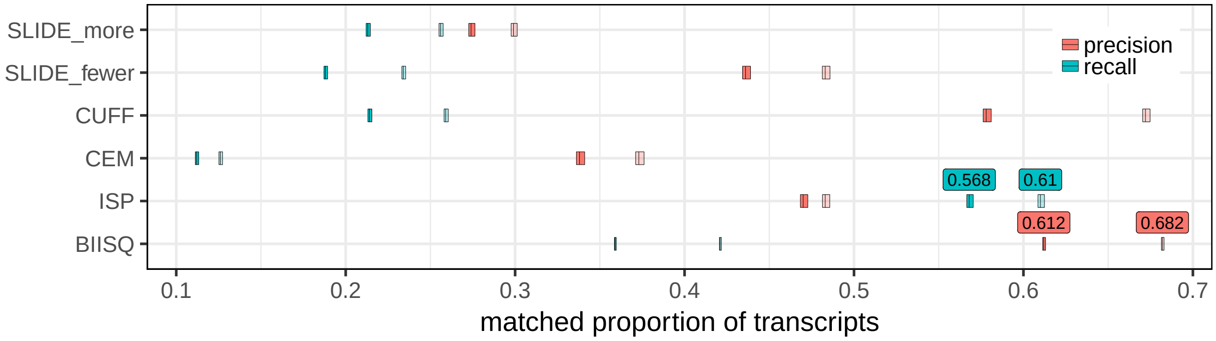

Supplementary Figure 3: **Isoform discovery precision and recall for central exons in BEERS simulated data.** Precision (red) and recall (blue) of the results from BIISQ, ISP, CEM, Cufflinks (CUFF), and SLIDE (SLIDE_more and SLIDE_fewer) applied to the BEERS simulated single-end RNA-seq data. The thick center bars denote the mean precision or recall and the fill denotes three times the standard error. Transparent fill denotes partial precision and recall with a matching threshold of 0.1. Across all methods, the best (partial) precision and recall values are annotated above their respective data points. We only consider exons which are terminal exons or between terminal exons of the true isoform.

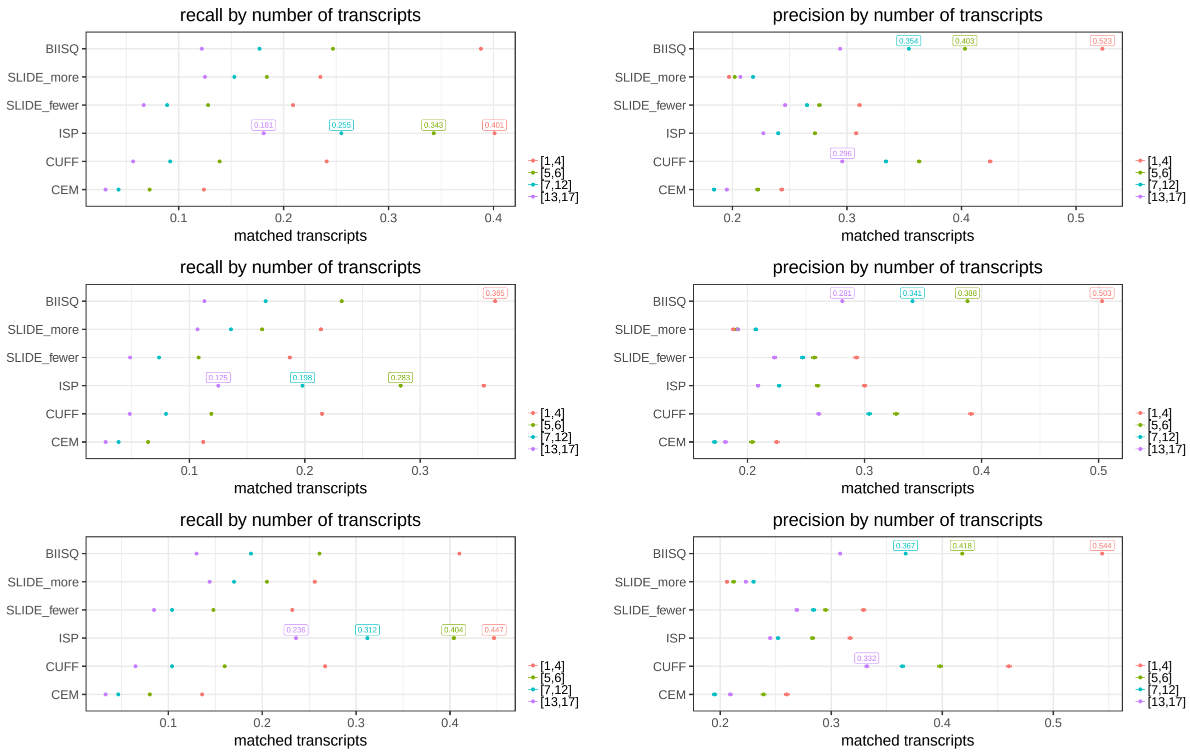

Supplementary Figure 4: **Recall and precision for BEERS data by number of transcripts.** (Left) Recall and (right) precision for BEERS data by number of transcripts per gene for (top row) both thresholds 0.0 and 0.1, (middle row) threshold 0.0 and (bottom row) threshold 0.1.

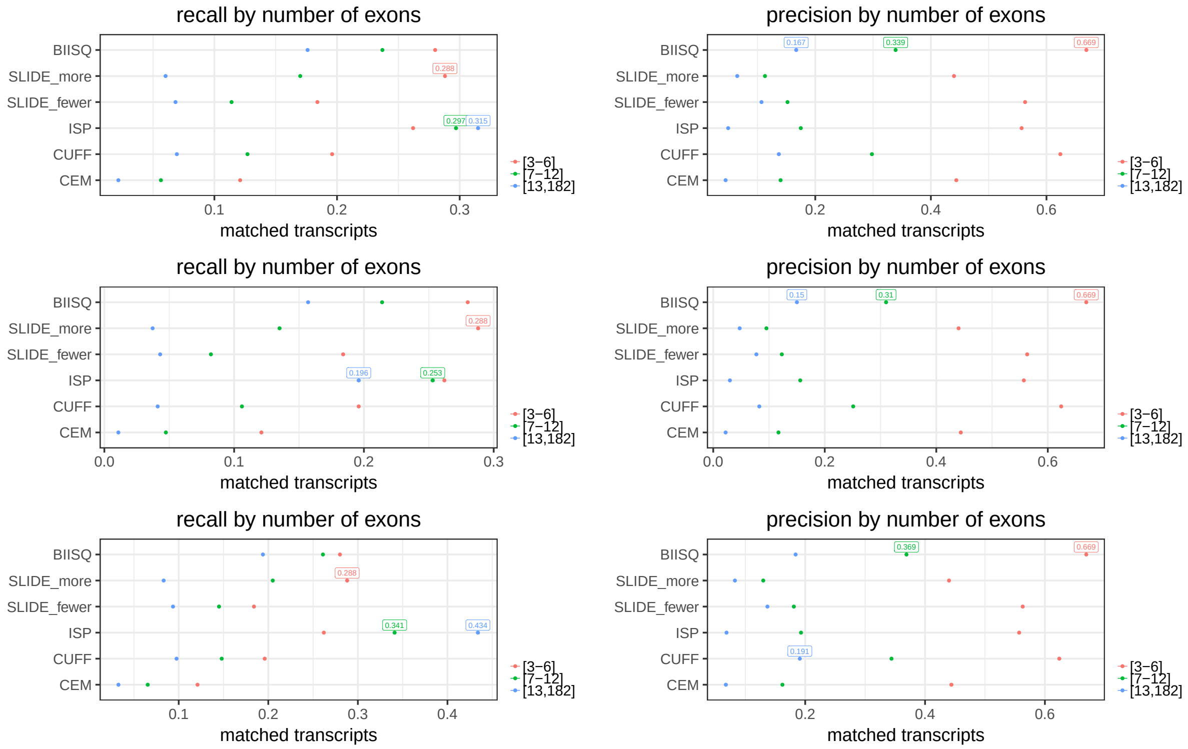

Supplementary Figure 5: **Recall and precision for BEERS data by number of exons.** (Left) Recall and (right) precision for BEERS data by number of exons per gene for (top row) both thresholds 0.0 and 0.1, (middle row) threshold 0.0 and (bottom row) threshold 0.1.

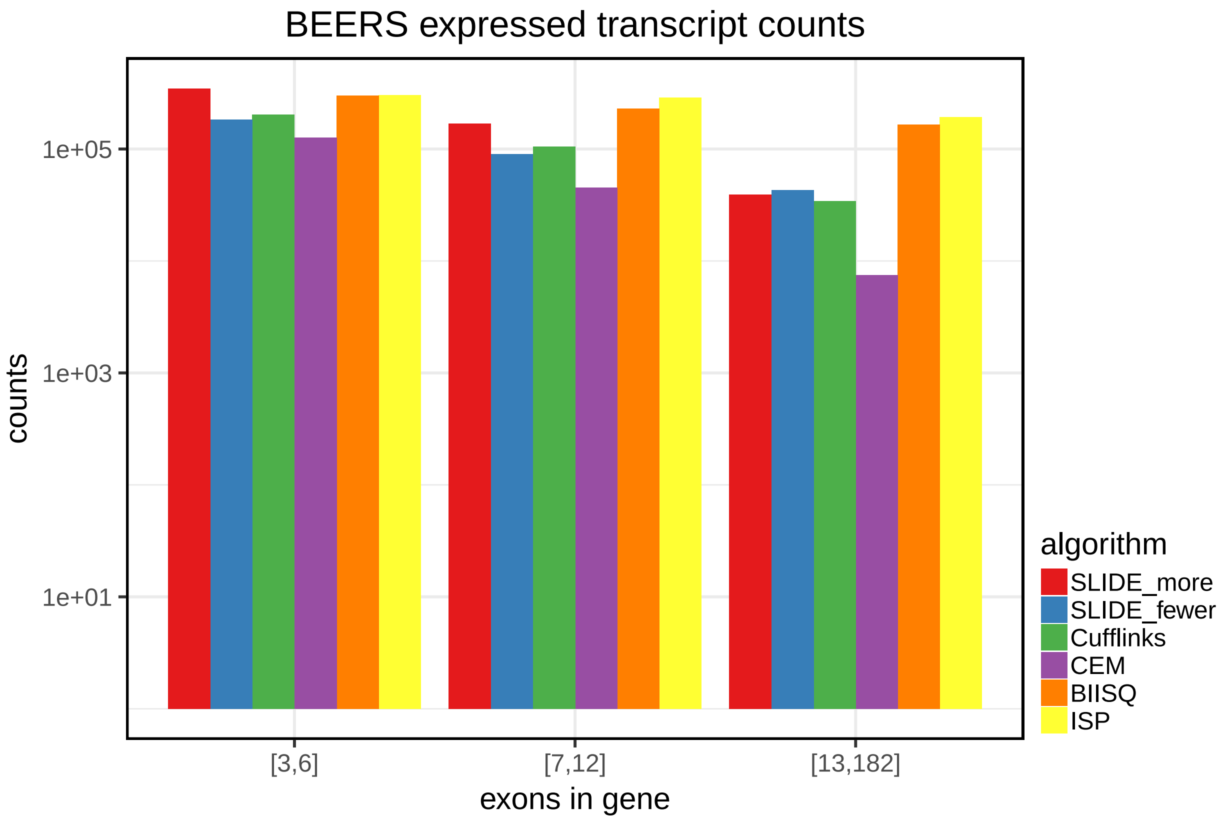

Supplementary Figure 6: **The number of correctly inferred and expressed transcripts in BEERS data by exon composition.** Genes are divided into three roughly equally sized groups (x-axis) and the number of transcripts inferred by each method is given on the y-axis. *Simulation* denotes the known simulated transcript baseline. SLIDE_more inferred at least $45,362$ more transcripts than other methods for transcripts where the number of exons was small (3-6 exons). However, BIISQ and *ISP* quantified $229,847$ and $288,460$ transcripts compared to the $169,387$ transcripts from the third best method, SLIDE_more, for medium length transcripts (7-12 exons); the gap widened for larger transcripts (13-182 exons) where BIISQ and *ISP* each inferred $> 123,000$ more transcripts than the other methods.

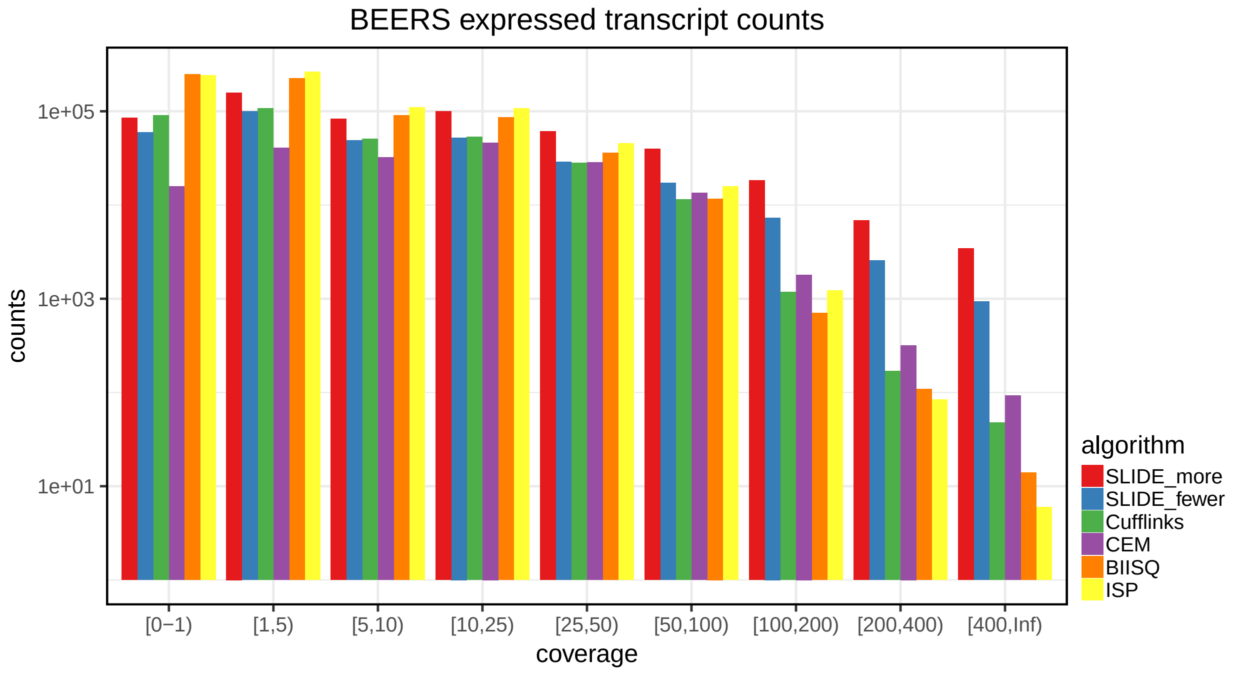

Supplementary Figure 7: **The number of correctly inferred and expressed transcripts in BEERS data by coverage.** BEERS simulated transcripts were divided into roughly equally sized groups based on coverage (x-axis). The number of inferred transcripts by each method is given on the y-axis. *Simulation* denotes the known simulated transcript baseline.

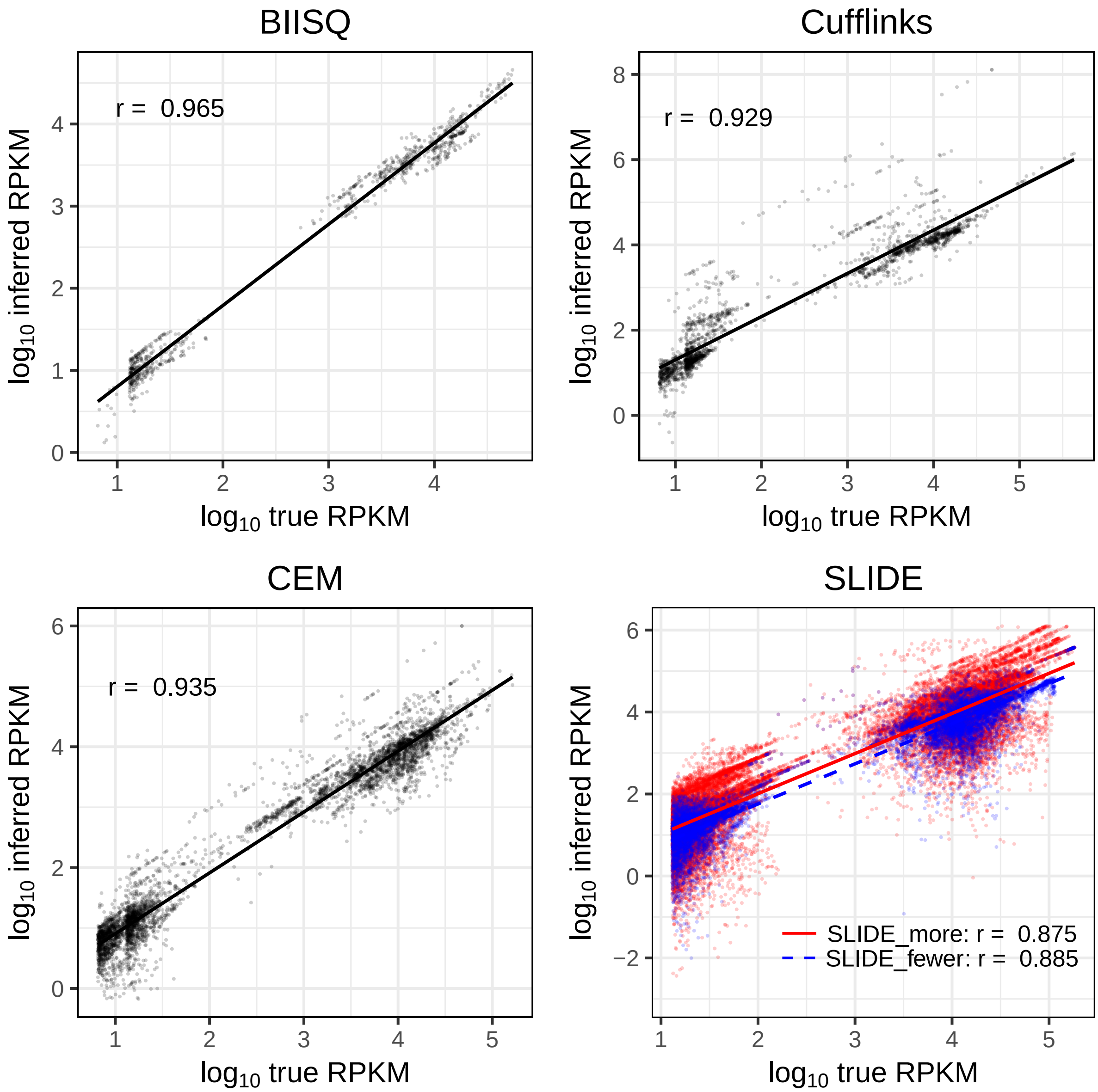

Supplementary Figure 8: **Isoform quantification accuracy on high coverage isoforms.** Correlation between true RPKM and inferred RPKM for BEERS simulated data high coverage > 100 transcripts. Spearman correlation coefficients for results from BIISQ, CEM, Cufflinks, SLIDE_more, and SLIDE_fewer were 0.965, 0.935, 0.929, 0.875, and 0.885, respectively, for BEERS simulated data. A regression line represents the best linear fit for each method to the expression data.

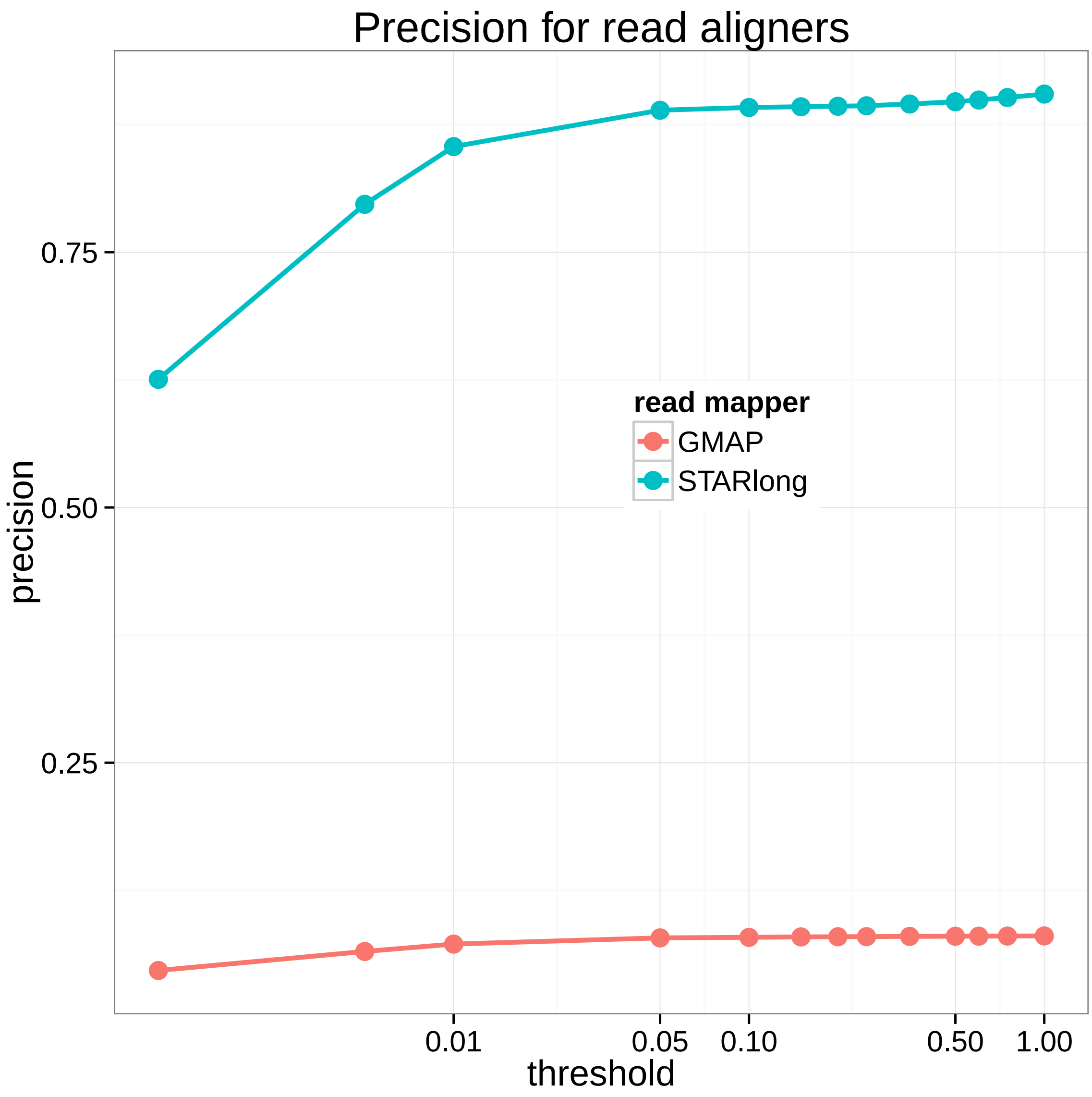

Supplementary Figure 9: **Read aligner performance (precision) on Iso-Seq data.** Full-length non-chimeric transcripts generated from PacBio Iso-Seq technology was mapped back to the whole human transcriptome from brain, heart, and liver tissue[1]. We mapped assembled transcripts to known annotations to reconstruct gene models for the eight interesting genes in Supplementary Table 2. The threshold denotes the percentage of non-clipped bases in the read that is allowed to map to non-exonic annotations. STARlong produces much fewer mappings, but with an order of magnitude higher precision.

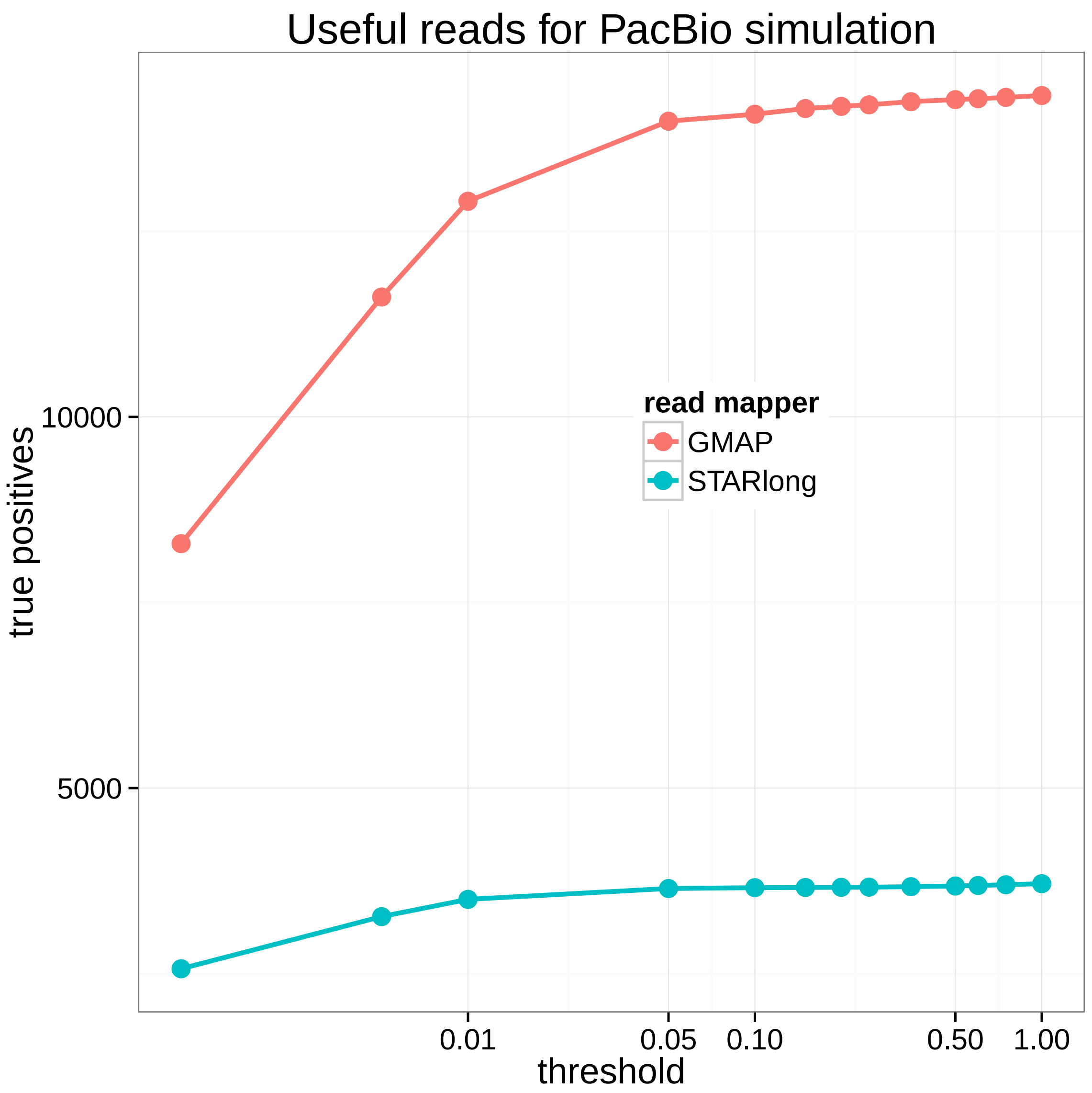

Supplementary Figure 10: **Read aligner performance (true positives) on Iso-Seq data.** Full-length non-chimeric transcripts generated from PacBio Iso-Seq technology was mapped back to the whole human transcriptome from brain, heart, and liver tissue[1]. We mapped assembled transcripts to known annotations to reconstruct gene models for the eight interesting genes in Supplementary Table 2. The threshold denotes the percentage of non-clipped bases in the read that is allowed to map to non-exonic annotations. While Supplementary Figure 9 shows superior precision for STARlong, the presence of annotation enables the filtering of true positives and false positives and GMAP produces more aligned true positives.

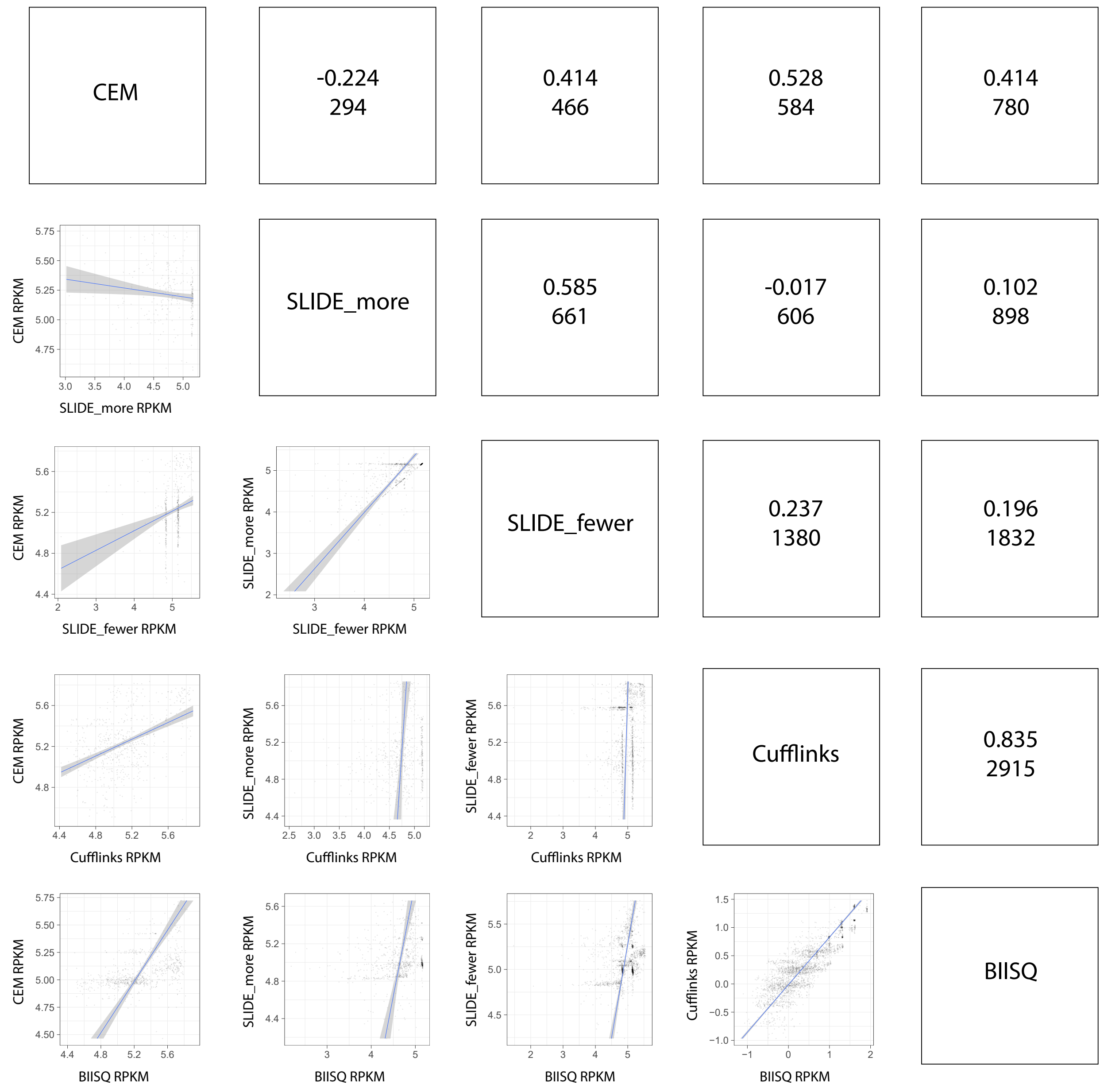

Supplementary Figure 11: **Correlation of expression values for shared transcripts across methods in the PacBio Iso-Seq data.** Correlation of inferred RPKM between the five methods are plotted where they intersect below the diagonal. Spearman *r* and the number of observations are given in the upper diagonal.

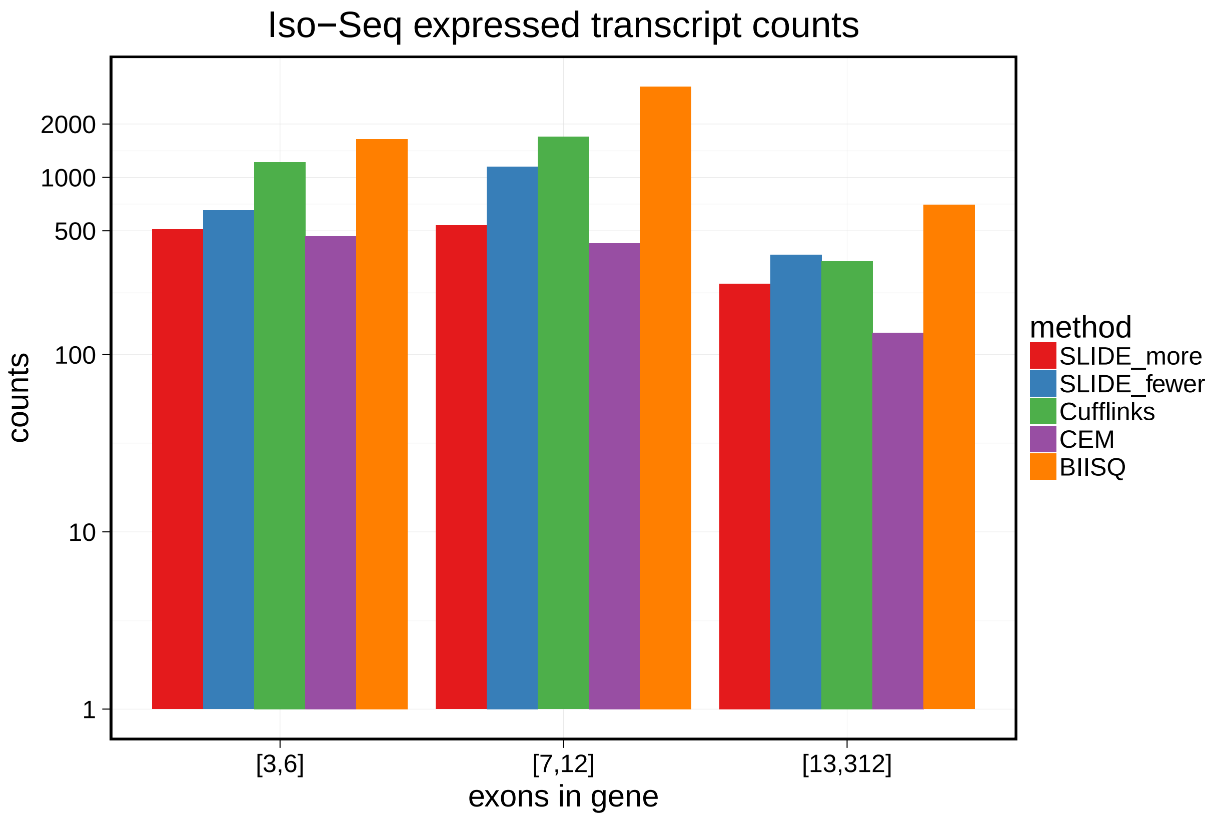

Supplementary Figure 12: **The number of correctly inferred and expressed transcripts in Iso-Seq data by exon composition.** Genes are divided into three roughly equally sized groups (x-axis) and the number of transcripts inferred by each method is given on the y-axis.

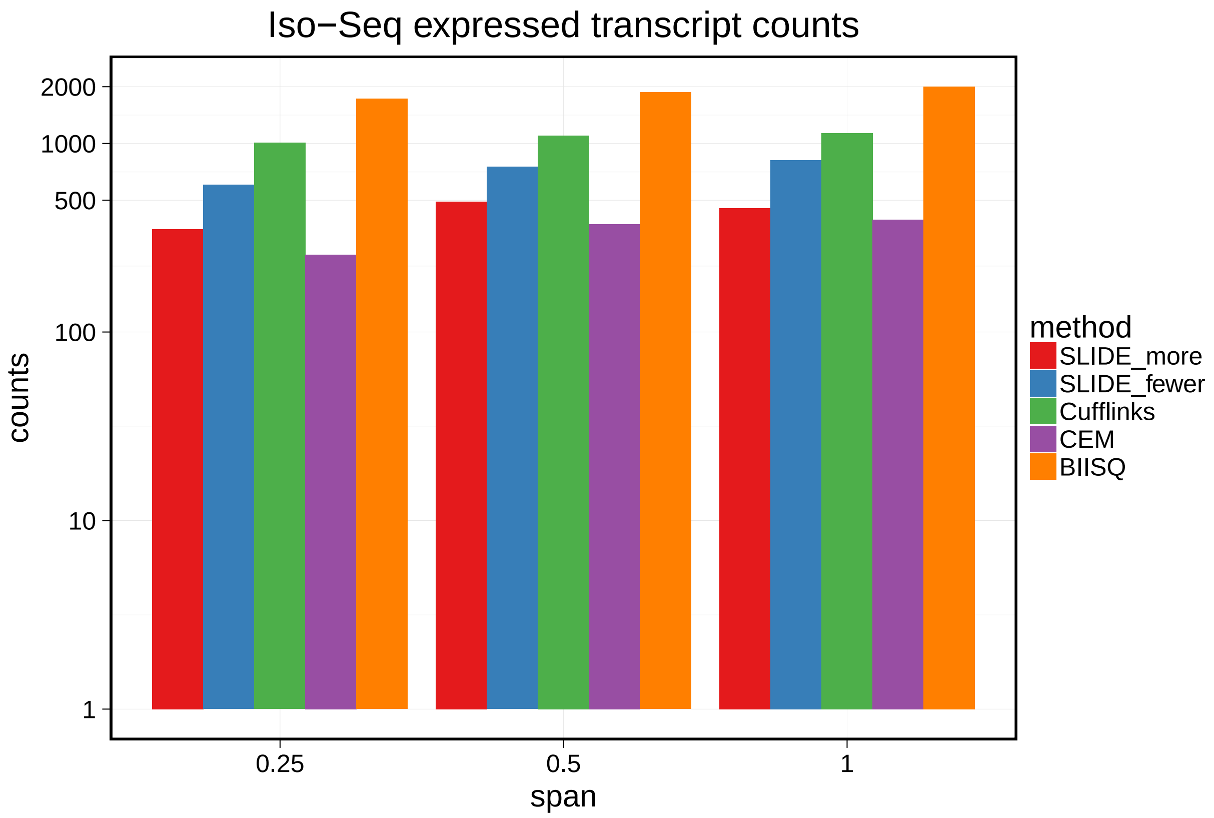

Supplementary Figure 13: **The number of correctly inferred and expressed transcripts in Iso-Seq data by span.** Iso-Seq generated transcripts were divided into roughly equally sized groups based on span (x-axis). The number of inferred transcripts by each method is given on the y-axis.

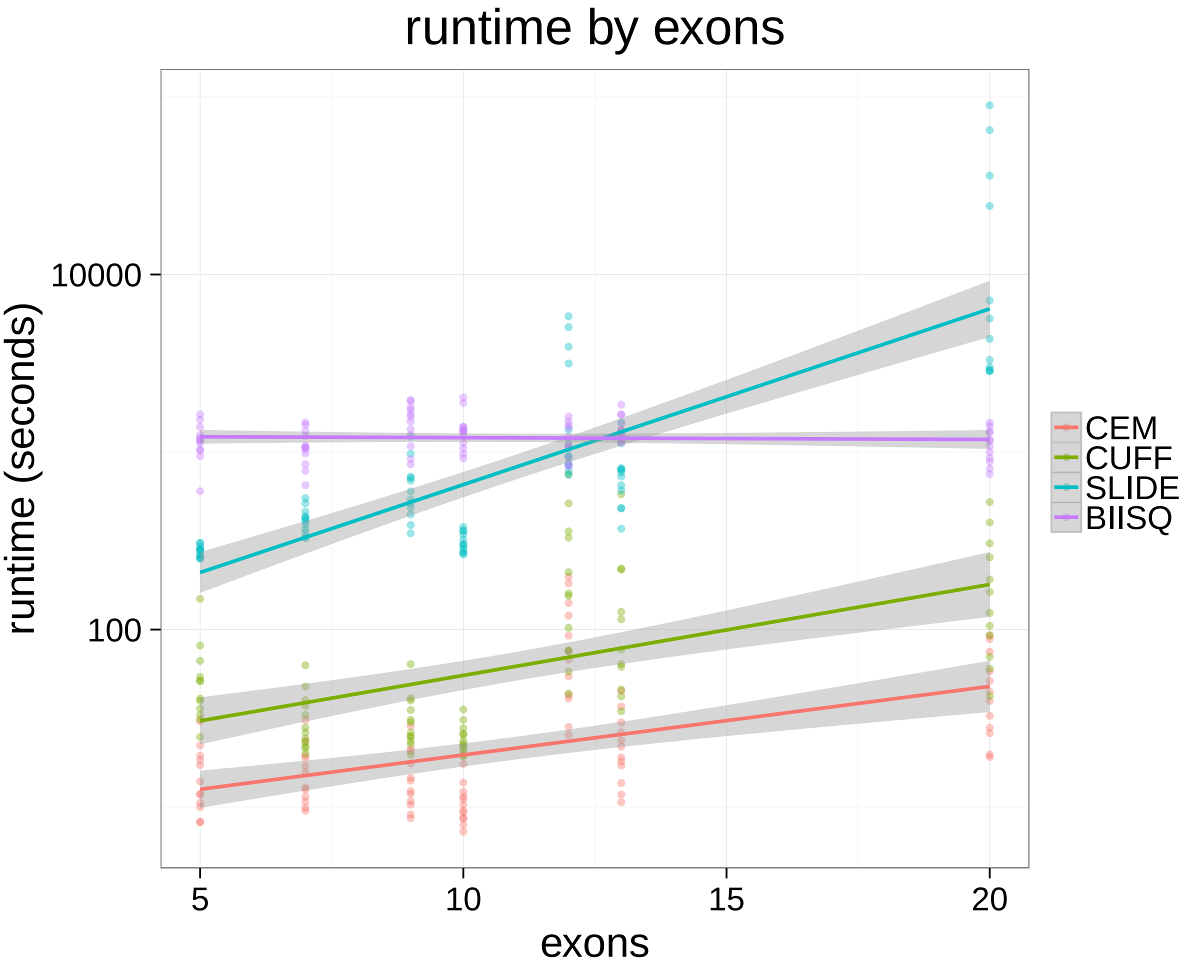

Supplementary Figure 14: **Method runtimes as a function of the number of exons on Iso-Seq data.** Runtime is measured in seconds and BIISQ runtimes are an average across 20 runs and do not include the one-time conversion of aligned reads to read terms. CEM and Cufflinks showed increased run time as a function of the number of exons (linear regression slope p-value for CEM and Cufflinks, $p \leq 8.79 \times 10^{-4}$ and $p \leq 1.64 \times 10^{-3}$, respectively). SLIDE_all showed increased run time as a function of exons ($p \leq 6.16 \times 10^{-6}$). The run time of BIISQ is not associated with the number of exons (linear regression slope p-value, $p \leq 0.61$).

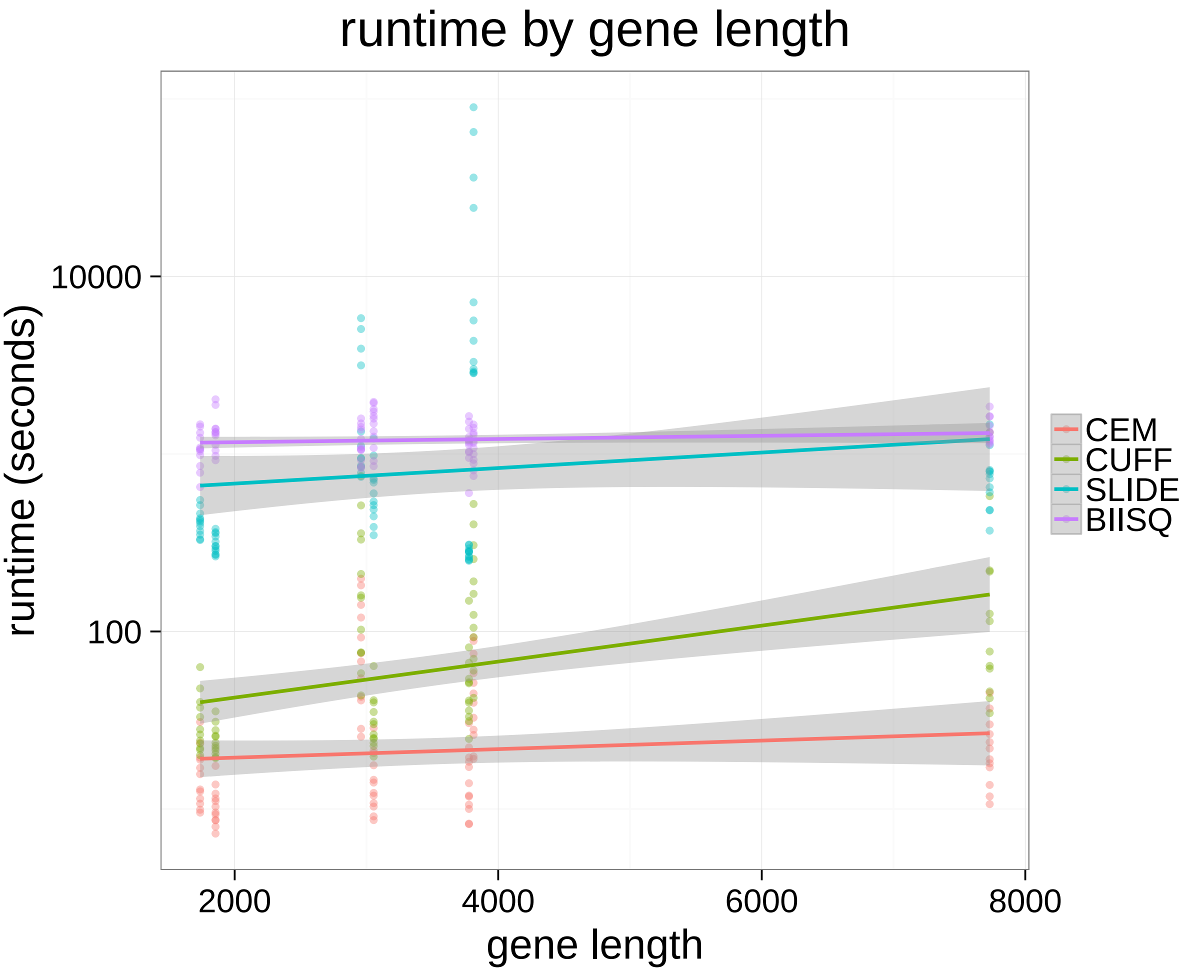

Supplementary Figure 15: **Method runtimes as a function of gene length on Iso-Seq data.** Runtime is measured in seconds and BIISQ runtimes are an average across 20 runs and do not include the one-time conversion of aligned reads to read terms.

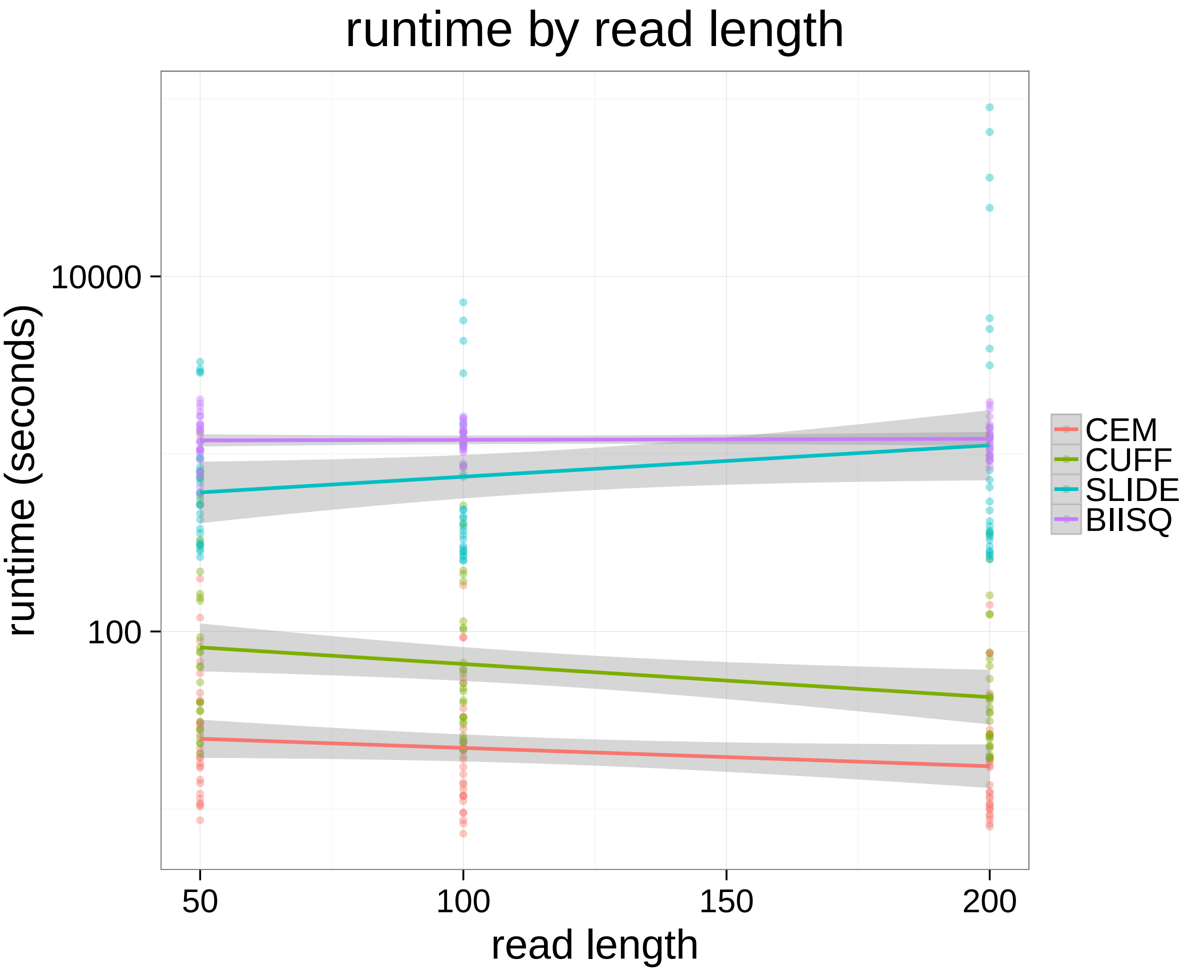

Supplementary Figure 16: **Method runtimes as a function of read length on Iso-Seq data.** Runtime is measured in seconds and BIISQ runtimes are an average across 20 runs and do not include the one-time conversion of aligned reads to read terms.

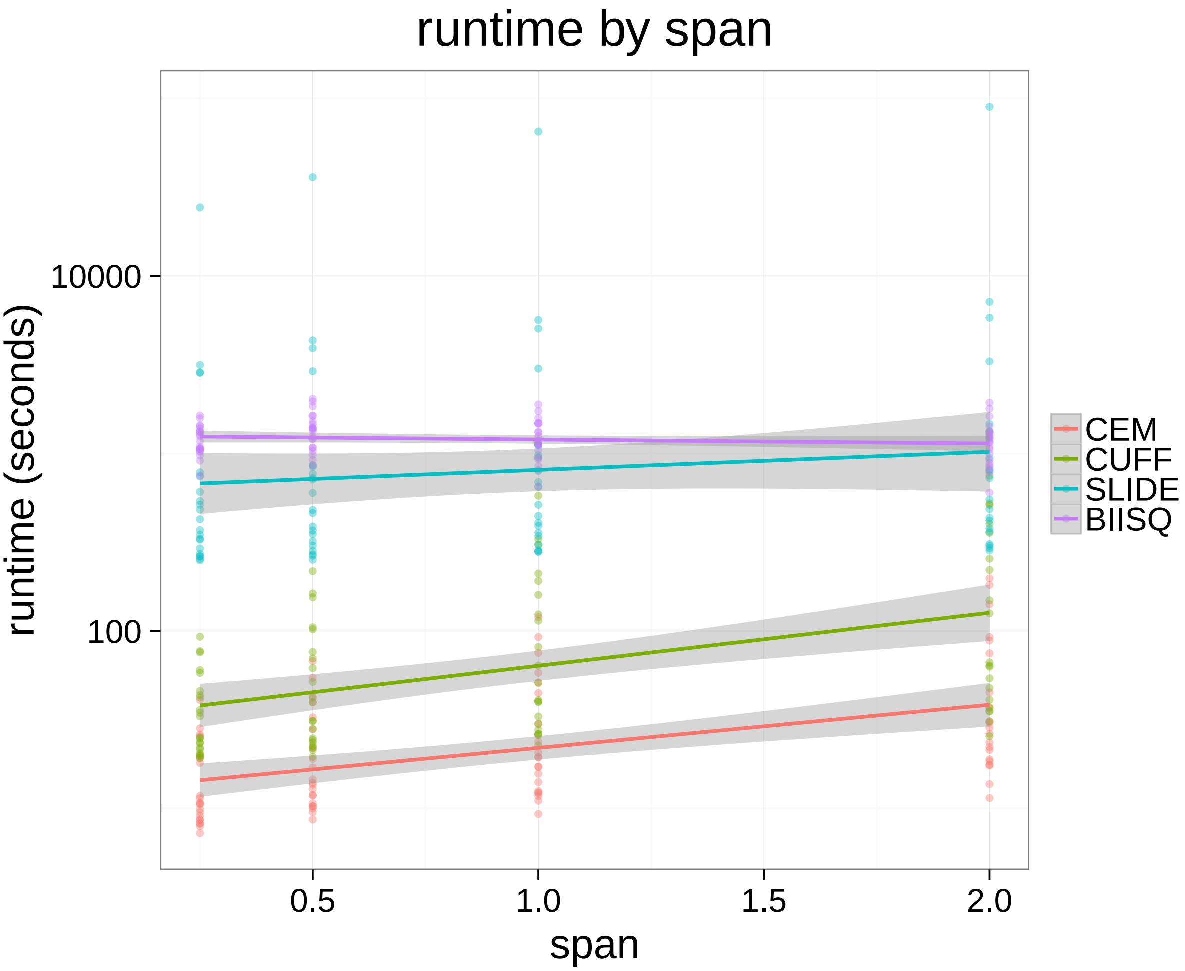

Supplementary Figure 17: **Method runtimes as a function of span on Iso-Seq data.** Runtime is measured in seconds and BIISQ runtimes are an average across 20 runs and do not include the one-time conversion of aligned reads to read terms. CEM and Cufflinks showed increased run time as a function of the span (linear regression slope p-value for CEM and Cufflinks, $p \leq 7.44 \times 10^{-5}$ and $p \leq 8.85 \times 10^{-5}$, respectively). The run time of BIISQ is not associated with the span (linear regression slope p-value, $p \leq 0.29$).

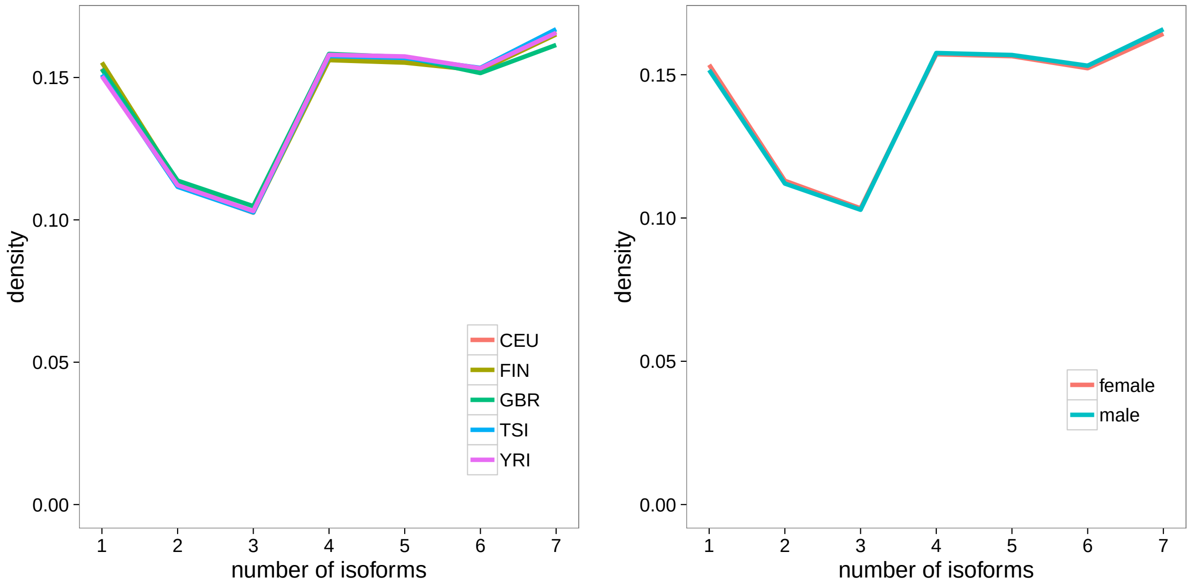

Supplementary Figure 18: **The density of genes with varying numbers of alternative isoforms by (left) population and (right) sex.**

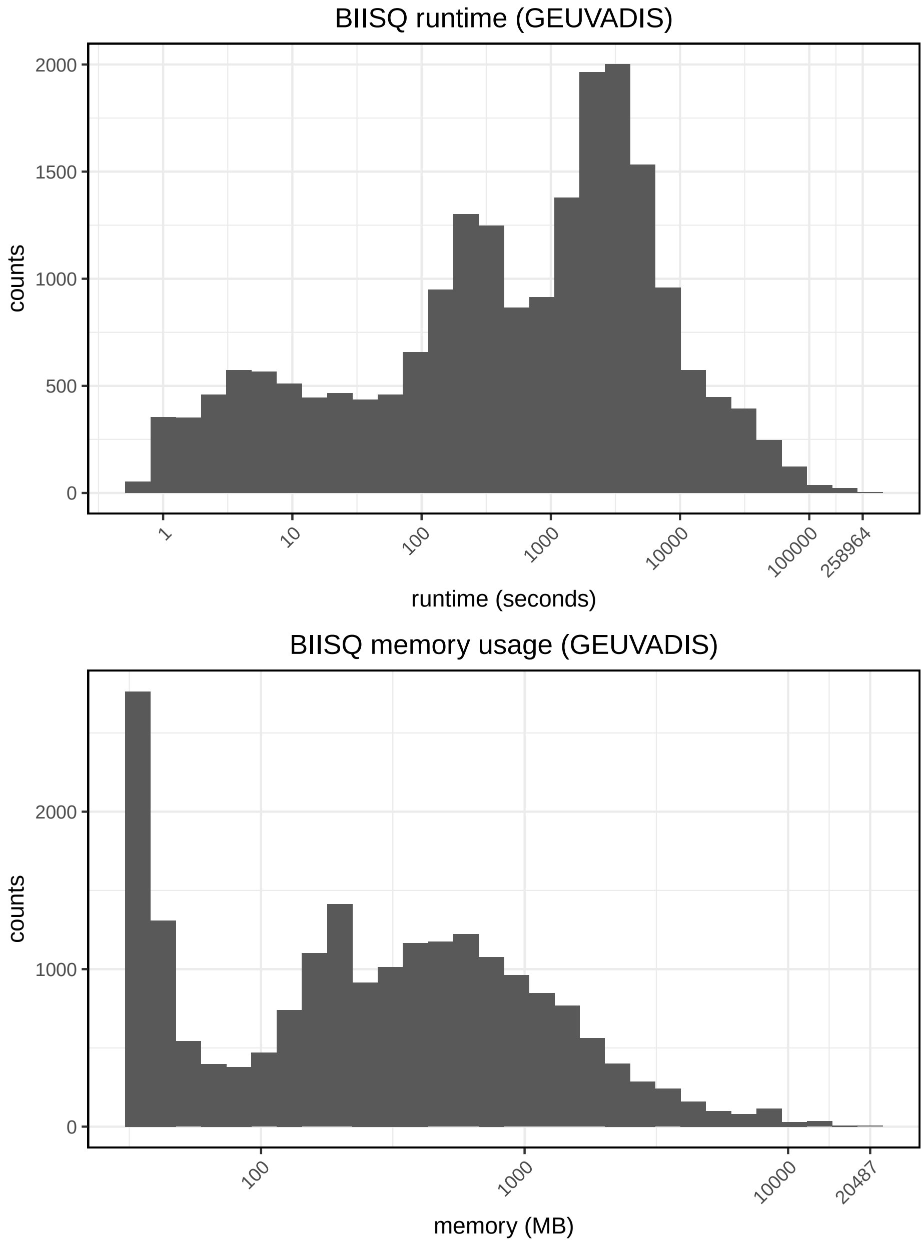

Supplementary Figure 19: **Histogram of BIISQ runtimes and memory usage for GEUVADIS data.** The runtime (top) and memory usage (bottom) of GEUVADIS jobs are counted on a logarithmic scale (x). The mean runtime and memory usage for BIISQ across the 20, 282 genes tested was 73.13 minutes and 700 MB, respectively, with a peak runtime of 71.93 hours and memory usage of 20.5 GB.

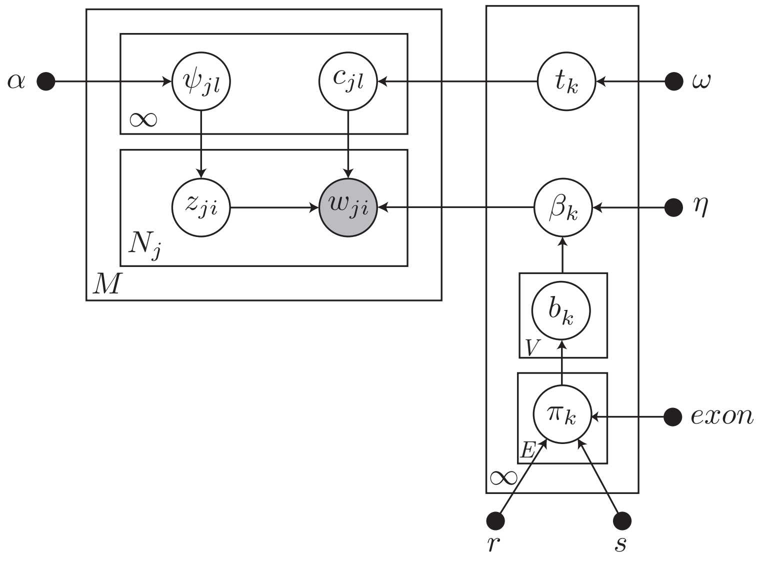

Supplementary Figure 20: **A graphical model for the generation of reads in RNA-seq data from M samples.** The left block denotes the sample-specific isoform distribution, and the right block denotes the population isoform distribution. The shaded circle denotes the observations (i.e., mapped reads), and the white circles denote the latent variables. The small solid circles on the sides denote the fixed hyperparameters.

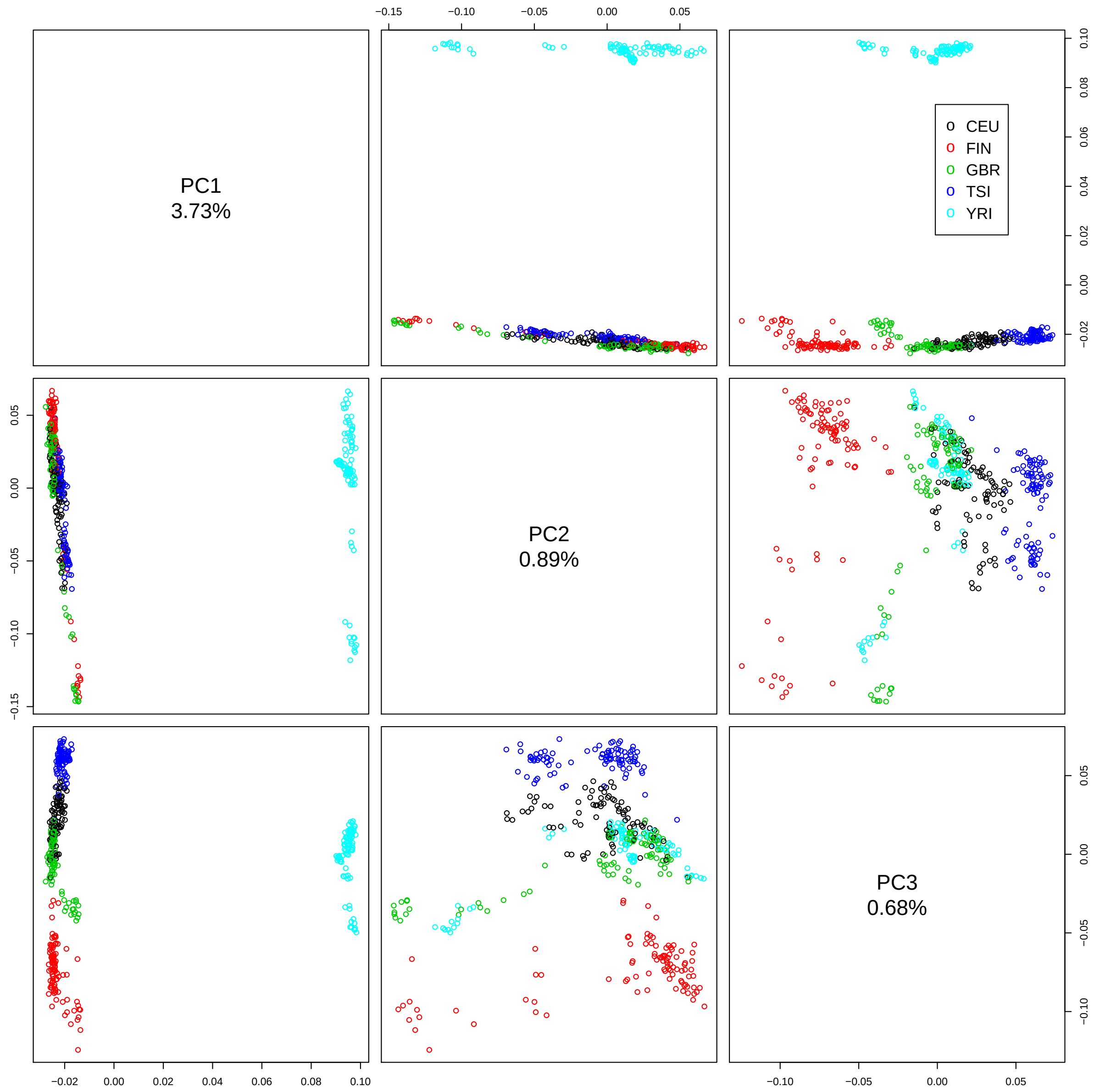

Supplementary Figure 21: **Genotype principal components (PCs) for Geuvadis data.** Population groups in the GEUVADIS data cluster well given the first three genotype PCs, which are used as covariates in the cis-trQTL linear regression model to correct for population structure.

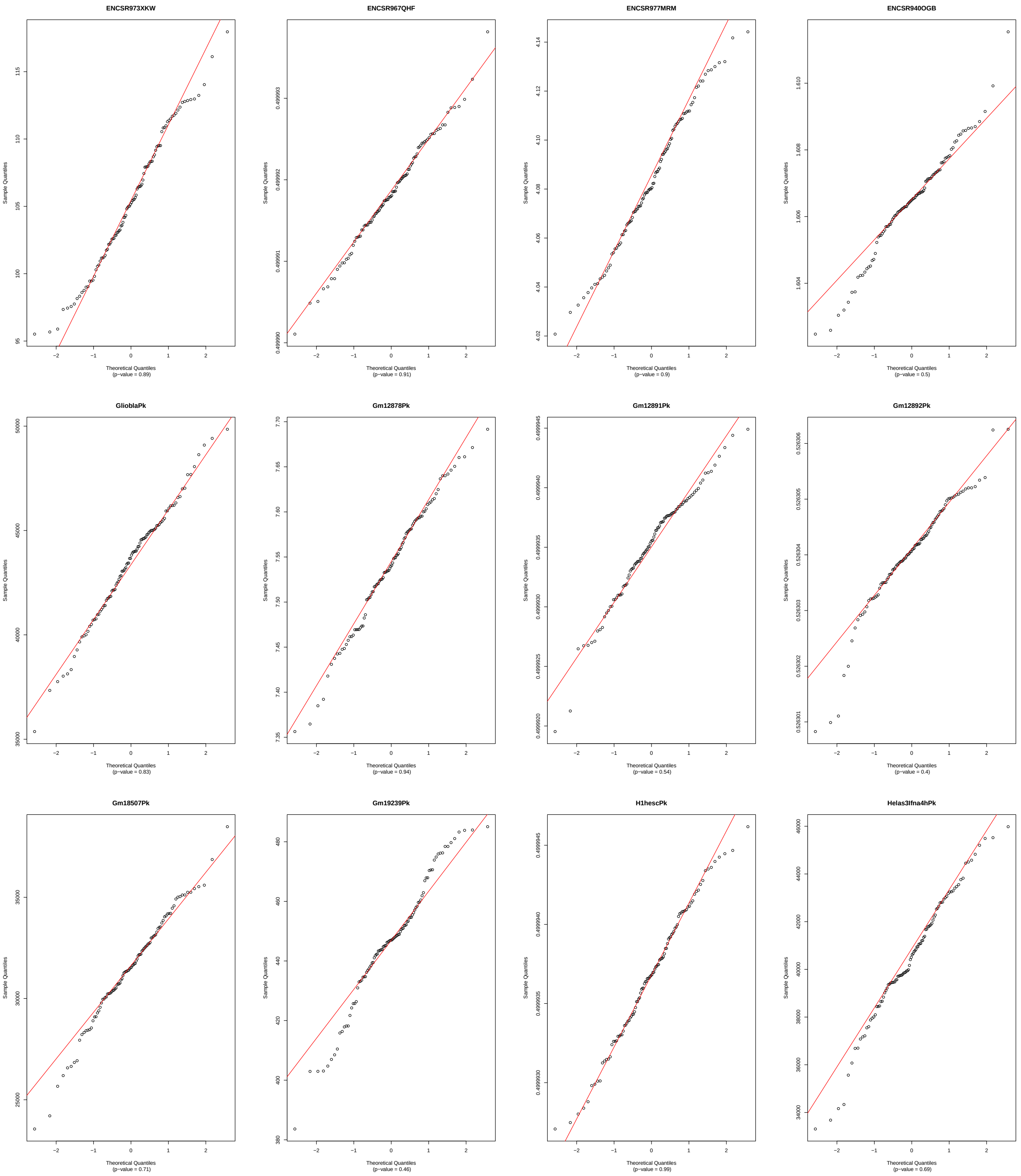
ENCSR973XKW
Sample Quantiles
Theoretical Quantiles
(p−value = 0.89)
ENCSR967QHF
Sample Quantiles
Theoretical Quantiles
(p−value = 0.91)
ENCSR977MRM
Sample Quantiles
Theoretical Quantiles
(p−value = 0.9)
ENCSR940OGB
Sample Quantiles
Theoretical Quantiles
(p−value = 0.5)
GlioblaPk
Sample Quantiles
Theoretical Quantiles
(p−value = 0.83)
Gm12878Pk
Sample Quantiles
Theoretical Quantiles
(p−value = 0.94)
Gm12891Pk
Sample Quantiles
Theoretical Quantiles
(p−value = 0.54)
Gm12892Pk
Sample Quantiles
Theoretical Quantiles
(p−value = 0.4)
Gm18507Pk
Sample Quantiles
Theoretical Quantiles
(p−value = 0.71)
Gm19239Pk
Sample Quantiles
Theoretical Quantiles
(p−value = 0.46)
H1hescPk
Sample Quantiles
Theoretical Quantiles
(p−value = 0.99)
Helas3Ifna4hPk
Sample Quantiles
Theoretical Quantiles
(p−value = 0.69)

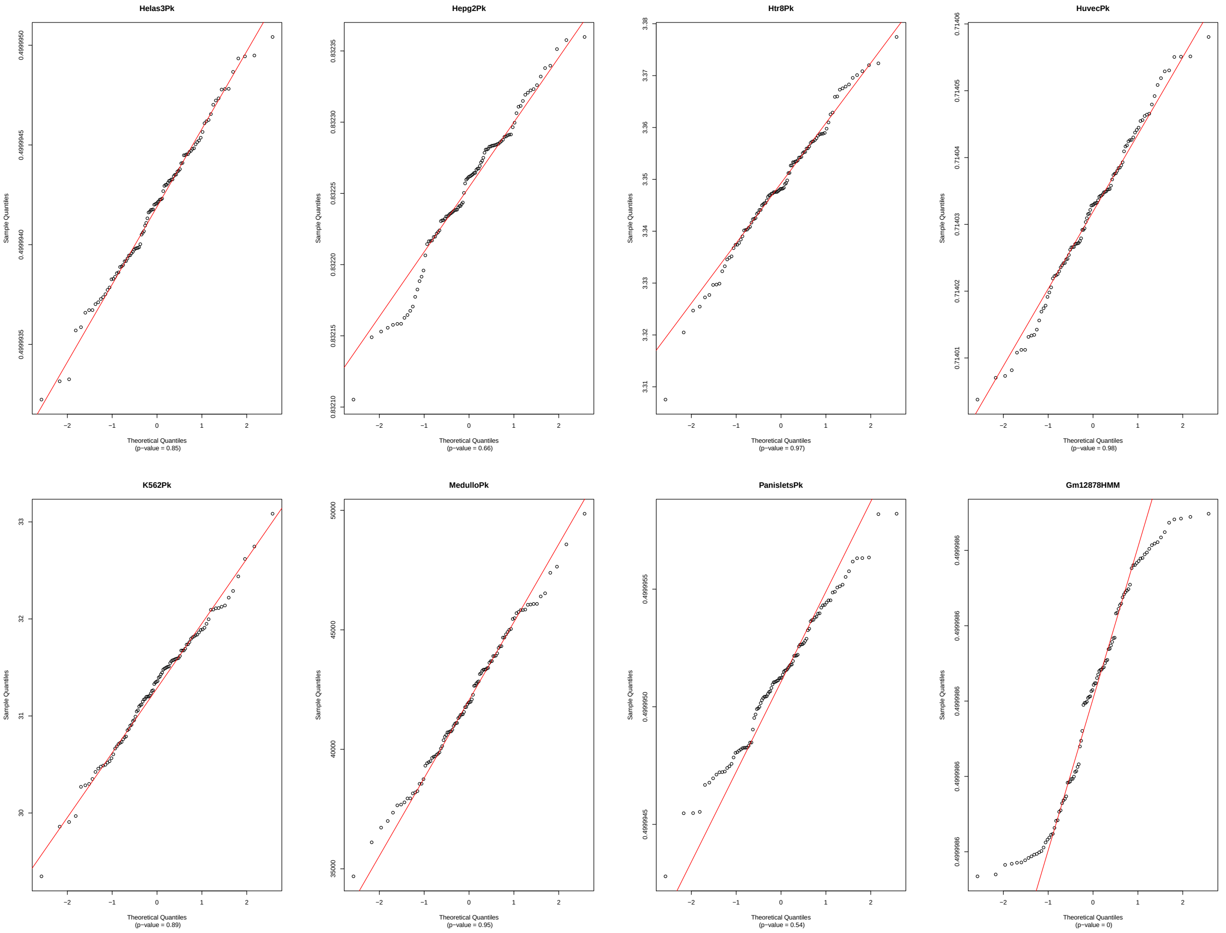

Supplementary Figure 22: **Theoretical versus sample quantiles for variant enrichment.** Only sample Gm12878HMM deviates significantly from normal (Kolmogorv-Smirnov test, $p \leq 2.2 \times 10^{-16}$).

Supplementary Table 1: **Exon coverage results for BEERS data.** TEC: Mean true exon coverage (IEC: Inferred exon coverage) for matched transcripts with a threshold of 0.0 or 0.1; TEC^{*}: Mean true exon coverage (IEC^{*}: Inferred exon coverage) for matched transcripts where exons outside of terminal vertices are not considered in distance calculations with a threshold of 0.0 or 0.1.

Method	TEC	TEC^{*}	IEC	IEC^{*}
BIISQ	0.916	0.968	0.916	0.968
CEM	0.283	0.410	0.285	0.411
Cufflinks	0.553	0.748	0.557	0.751
ISP	0.774	0.857	0.781	0.863
SLIDE_fewer	0.531	0.714	0.533	0.717
SLIDE_more	0.606	0.665	0.610	0.670

Supplementary Table 2: **Iso-Seq gold standard.** Columns include internal ID, chromosome, transcription start site (TSS), transcription end site (TES), and gene symbol.

internal ID	Chr	TSS	TES	gene symbol
$IDISQ\_3426$	chr15	45879574	45901903	*BLOC1S6*
$IDISQ\_3491$	chr15	80352035	80430726	*ZFAND6*
$IDISQ\_3930$	chr17	76670132	76778389	*CYTH1*
$IDISQ\_4630$	chr21	27252863	27543410	*APP*
$IDISQ\_4902$	chr1	154179184	154193113	*C1orf43*
$IDISQ\_5157$	chr4	88394490	88450618	*SPARCL1*
$IDISQ\_5405$	chr5	141348670	141368742	*RNF14*

Supplementary Table 3: **Variant enrichment sample descriptions.** The samples described as *DNaseI/FAIRE/ChIP Synthesis* are a combination of open chromatin regions and transcription factor binding sites from DNaseI hypersensitivity (HS), Formaldehyde-Assisted Isolation of Regulatory Elements (FAIRE), and chromatin immunoprecipitation (ChIP) for regulatory factors described by the ENCODE project[2].

Sample ID	Accession	Cell type	Description	Ref
Bcell-1	ENCSR973XKW	B cell	Promoter-like regions using DNase and H3K4me3 for B cell	2
Bcell-2	ENCSR967QHF	B cell	Enhancer-like regions using DNase-only for B cell	2
Bcell-3	ENCSR977MRM	B cell	Enhancer-like regions using DNase and H3K27ac for B cell	2
Bcell-4	ENCSR940OGB	B cell	Enhancer-like regions using DNase-only for B cell	2
LCL-1	GSM936082	B-lymphocyte	Chromatin State Segmentation, Multi-assay Synthesis. Source data from ChIP-seq data from the Broad Histone UCSC track for nine factors and nine cell types.	3
LCL-2	ENCSR918FQM	B-lymphocyte	DNaseI/FAIRE/ChIP Synthesis from ENCODE/OpenChrom(Duke/UNC/UTA)	2
LCL-3	ENCSR601DAI	B-lymphocyte	DNaseI/FAIRE/ChIP Synthesis from ENCODE/OpenChrom(Duke/UNC/UTA)	2
LCL-4	ENCSR893EGV	B-lymphocyte	DNaseI/FAIRE/ChIP Synthesis from ENCODE/OpenChrom(Duke/UNC/UTA)	2
LCL-5	ENCSR273KTY	B-lymphocyte	DNaseI/FAIRE/ChIP Synthesis from ENCODE/OpenChrom(Duke/UNC/UTA)	2
LCClusters-1	No Accession	LeafCutter	DNaseI/FAIRE/ChIP Synthesis from ENCODE/OpenChrom(Duke/UNC/UTA)	2
Glioblast-1	ENCSR934HWL	glioblastoma	the H54 and D54 cells come from surgical resection froma glioblastoma multiforme patient. DNaseI/FAIRE/ChIP Synthesis from ENCODE/OpenChrom(Duke/UNC/UTA)	2
EStemCell-1	ENCSR236BTH	embryonic stem cell	DNaseI/FAIRE/ChIP Synthesis from ENCODE/OpenChrom(Duke/UNC/UTA)	2
CervicalC-1	ENCSR563SKA	cervical carcinoma	DNaseI/FAIRE/ChIP Synthesis from ENCODE/OpenChrom(Duke/UNC/UTA)	2
CervicalC-2	ENCSR236YNV	cervical carcinoma	DNaseI/FAIRE/ChIP Synthesis from ENCODE/OpenChrom(Duke/UNC/UTA)	2
HepatoC-1	ENCSR283COF	hepatocellular carcinoma	DNaseI/FAIRE/ChIP Synthesis from ENCODE/OpenChrom(Duke/UNC/UTA)	2
TrophoB-1	ENCSR066RYF	trophoblast	DNaseI/FAIRE/ChIP Synthesis from ENCODE/OpenChrom(Duke/UNC/UTA)	2
Endothelial-1	ENCSR518LJU	umbilical vein endothelial cell	DNaseI/FAIRE/ChIP Synthesis from ENCODE/OpenChrom(Duke/UNC/UTA)	2
MLeukemia-1	ENCSR122HHF	mesoderm leukemia	DNaseI/FAIRE/ChIP Synthesis from ENCODE/OpenChrom(Duke/UNC/UTA)	2
MedulloB-1	ENCSR237MJM	medulloblastoma	DNaseI/FAIRE/ChIP Synthesis from ENCODE/OpenChrom(Duke/UNC/UTA)	2
PancreasI-1	ENCSR441NJL	pancreatic islets	DNaseI/FAIRE/ChIP Synthesis from ENCODE/OpenChrom(Duke/UNC/UTA)	2

Supplementary Table 4: **Gene annotation categories used in enrichment analysis.**

abbreviation	description
UniProt tissue	literature mined annotations of gene expression specificity
InterPro	family and domain classifications for proteins from InterPro
KEGG	pathway annotations created from the KEGG database
GO-BP	biological processes gene ontology annotations
GO-CC	cellular component gene ontology annotations
GO-MF	molecular function gene ontology annotations
SwissProt	SwissProt or Uniprot and the Protein Information Resource keywords
UniProt seq-feature	the annotation category “Uniprot Sequence Feature” that describes regions or sites of interest for proteins
OMIM	gene annotations built from the Online Mendelian Inheritance in Man NCBI database

Supplementary Table 5: **Functional enrichment for genes with at least one trQTL.**

Category	Term	Count	List Total	Pop Hits	Pop Total	Fold Enrichment	PValue	Bonferroni	BH FDR
UP_SEQ_FEATURE	sequence variant	470	649	12444	20064	1.167644607	1.10E-08	2.09E-05	2.09E-05
UP_KEYWORDS	Polymorphism	462	667	12023	20042	1.154634028	3.20E-07	1.26E-04	1.26E-04
KEGG_PATHWAY	hsa04740:Olfactory transduction	33	246	399	6891	2.316798093	1.06E-05	0.002488606	0.002488606
UP_KEYWORDS	Olfaction	33	667	439	20042	2.258731682	3.02E-05	0.011839567	0.00593741
UP_KEYWORDS	Glycoprotein	194	667	4542	20042	1.283423018	6.84E-05	0.026572524	0.006710373
UP_KEYWORDS	Sensory transduction	40	667	604	20042	1.989932186	6.42E-05	0.024993107	0.008401422
UP_KEYWORDS	Membrane	282	667	7098	20042	1.193791101	1.38E-04	0.052776887	0.010785537
UP_SEQ_FEATURE	glycosylation site:N-linked (GlcNAc...)	180	649	4235	20064	1.313989554	3.65E-05	0.067165059	0.034166194
GOTERM_MF_DIRECT	GO:0004984 olfactory receptor activity	32	561	425	16483	2.212251232	5.72E-05	0.036114597	0.036114597
UP_KEYWORDS	Disulfide bond	144	667	3357	20042	1.288921223	7.64E-04	0.260071361	0.048961045

Supplementary Table 6: **Functional enrichment for isoforms with >1 transcripts.**

Category	Term	Count	List Total	Pop Hits	Pop Total	Fold Enrichment	PValue	Bonferroni	BH FDR
UP_KEYWORDS	Alternative splicing	9043	13804	10581	20042	1.240857539	0	0	0
UP_SEQ_FEATURE	splice variant	6546	13613	7761	20064	1.243145586	0	0	0
UP_KEYWORDS	Phosphoprotein	6275	13804	7493	20042	1.215889491	3.70E-287	2.65E-284	1.33E-284
UP_KEYWORDS	Proteomics identification	2125	13804	2405	20042	1.282862059	1.69E-124	1.21E-121	4.04E-122
UP_KEYWORDS	Cytoplasm	3907	13804	4750	20042	1.194224313	7.76E-124	5.56E-121	1.39E-121
UP_KEYWORDS	Acetylation	2912	13804	3426	20042	1.234070922	2.38E-123	1.71E-120	3.41E-121
GOTERM_MF_DIRECT	GO:0005515 protein binding	6994	11974	8706	16483	1.105869728	3.65E-122	1.27E-118	1.27E-118
GOTERM_CC_DIRECT	GO:0005829 cytosol	2719	12629	3217	17701	1.184641617	6.68E-82	9.53E-79	9.53E-79
UP_KEYWORDS	Nucleotide-binding	1540	13804	1774	20042	1.260384961	7.00E-75	5.02E-72	8.36E-73
UP_KEYWORDS	ATP-binding	1230	13804	1384	20042	1.290342876	3.99E-73	2.86E-70	4.09E-71
UP_KEYWORDS	Coiled coil	2190	13804	2649	20042	1.200323375	1.18E-66	8.49E-64	1.06E-64
GOTERM_CC_DIRECT	GO:0005654 nucleoplasm	2173	12629	2561	17701	1.189265955	1.80E-66	2.57E-63	1.28E-63
GOTERM_CC_DIRECT	GO:0005737 cytoplasm	3855	1.26E+04	4.80E+03	17701	1.125906876	1.35E-61	1.93E-58	6.43E-59
UP_TISSUE	Brain	6275	13204	8256	18913	1.088676762	4.14E-61	2.62E-58	2.62E-58
UP_KEYWORDS	Polymorphism	8797	1.38E+04	1.20E+04	20042	1.062326101	3.79E-58	2.72E-55	3.02E-56
UP_SEQ_FEATURE	sequence variant	8956	13613	12444	20064	1.06076152	2.08E-57	4.47E-53	2.23E-53
GOTERM_CC_DIRECT	GO:0016020 membrane	1631	1.26E+04	1.90E+03	17701	1.202543189	2.86E-55	4.08E-52	1.02E-52
UP_KEYWORDS	Metal-binding	2886	1.38E+04	3.64E+03	20042	1.1511477	4.37E-54	3.14E-51	3.14E-52
UP_SEQ_FEATURE	nucleotide phosphate-binding region:ATP	882	13613	994	20064	1.307813679	1.41E-55	3.04E-51	1.01E-51
GOTERM_MF_DIRECT	GO:0005524 ATP binding	1301	11974	1467	16483	1.220799064	8.49E-55	2.96E-51	1.48E-51
UP_KEYWORDS	Cytoskeleton	970	13804	1112	20042	1.266493759	1.78E-48	1.28E-45	1.16E-46
UP_KEYWORDS	Disease mutation	1987	13804	2464	20042	1.170828461	1.20E-44	8.63E-42	7.19E-43
UP_KEYWORDS	Ubl conjugation	1373	1.38E+04	1.67E+03	20042	1.195834406	7.31E-39	5.24E-36	4.03E-37
UP_TISSUE	Epithelium	2251	13204	2816	18913	1.144979607	1.82E-39	1.15E-36	5.77E-37
UP_KEYWORDS	Nucleus	3932	13804	5183	20042	1.101459182	4.22E-38	3.03E-35	2.16E-36
UP_KEYWORDS	Transferase	1335	13804	1627	20042	1.191323805	3.53E-36	2.53E-33	1.69E-34
UP_KEYWORDS	Kinase	628	13804	709	20042	1.286025309	7.03E-36	5.04E-33	3.15E-34
UP_SEQ_FEATURE	mutagenesis site	1742	1.36E+04	2.19E+03	20064	1.171843048	7.21E-38	1.55E-33	3.88E-34
UP_KEYWORDS	Hydrolase	1346	13804	1653	20042	1.182247253	2.15E-33	1.54E-30	9.05E-32
UP_SEQ_FEATURE	active site:Proton acceptor	587	1.36E+04	6.72E+02	20064	1.287456318	2.12E-32	4.57E-28	9.14E-29
UP_SEQ_FEATURE	binding site:ATP	491	13613	558	20064	1.296913371	6.79E-29	1.46E-24	2.44E-25
UP_SEQ_FEATURE	domain:Protein kinase	427	1.36E+04	4.78E+02	20064	1.316629717	1.78E-28	3.84E-24	5.49E-25
UP_KEYWORDS	Transport	1553	13804	1965	20042	1.147479692	1.89E-26	1.36E-23	7.55E-25
UP_KEYWORDS	Zinc	1844	13804	2363	20042	1.13300885	2.69E-26	1.93E-23	1.01E-24
UP_KEYWORDS	Cell cycle	554	13804	640	20042	1.256799207	6.92E-26	4.96E-23	2.48E-24
UP_KEYWORDS	Protein transport	527	13804	606	20042	1.262624169	1.05E-25	7.56E-23	3.60E-24
UP_KEYWORDS	Magnesium	482	13804	550	20042	1.272390611	3.18E-25	2.28E-22	1.04E-23
GOTERM_CC_DIRECT	GO:0005739 mitochondrion	1037	1.26E+04	1.24E+03	17701	1.174050967	4.18E-26	5.95E-23	1.19E-23
UP_KEYWORDS	Cell projection	575	13804	675	20042	1.236802	3.87E-23	2.77E-20	1.21E-21
INTERPRO	IPR011009:Protein kinase-like domain	474	12994	530	18205	1.252997755	3.45E-24	2.72E-20	1.36E-20
INTERPRO	IPR011993:Pleckstrin homology-like domain	390	1.30E+04	4.27E+02	18205	1.279630411	2.88E-24	2.26E-20	2.26E-20
UP_KEYWORDS	Zinc-finger	1413	1.38E+04	1.81E+03	20042	1.136582756	4.96E-21	3.56E-18	1.48E-19
UP_KEYWORDS	Cell division	337	13804	378	20042	1.294417001	1.20E-20	8.62E-18	3.45E-19
INTERPRO	IPR000719:Protein kinase, catalytic domain	435	12994	486	18205	1.254009448	2.00E-22	1.57E-18	5.24E-19
UP_KEYWORDS	Serine/threonine-protein kinase	350	13804	395	20042	1.286491899	2.38E-20	1.70E-17	6.56E-19
UP_KEYWORDS	Mitochondrion	890	13804	1103	20042	1.171522412	6.75E-20	4.84E-17	1.79E-18
INTERPRO	IPR027417:P-loop containing nucleoside triphosphate hydrolase	739	12994	871	18205	1.18870504	1.17E-21	9.18E-18	2.29E-18
UP_KEYWORDS	Chromatin regulator	262	13804	287	20042	1.325426049	1.37E-19	9.85E-17	3.52E-18

UP_KEYWORDS	Endoplasmic reticulum	854	1.38E+04	1.06E+03	20042	1.173056663	2.01E-19	1.44E-16	4.97E-18
UP_KEYWORDS	Isopeptide bond	815	13804	1008	20042	1.173905625	9.81E-19	7.04E-16	2.35E-17
UP_KEYWORDS	DNA damage	304	1.38E+04	3.41E+02	20042	1.294360681	1.06E-18	7.59E-16	2.45E-17
UP_TISSUE	Uterus	1439	13204	1833	18913	1.124483885	6.75E-19	4.27E-16	1.42E-16
GOTERM_CC_DIRECT	GO:0070062 extracellular exosome	2162	12629	2769	17701	1.094363432	8.60E-19	1.23E-15	2.04E-16
UP_KEYWORDS	Host-virus interaction	336	1.38E+04	3.84E+02	20042	1.270410751	1.29E-17	9.27E-15	2.90E-16
UP_KEYWORDS	WD repeat	253	13804	281	20042	1.30722489	5.36E-17	3.84E-14	1.16E-15
GOTERM_CC_DIRECT	GO:0005813 centrosome	369	12629	416	17701	1.243259752	1.17E-17	1.66E-14	2.37E-15
UP_TISSUE	Placenta	2687	13204	3558	18913	1.081724409	1.56E-17	9.85E-15	2.46E-15
UP_KEYWORDS	Golgi apparatus	657	13804	806	20042	1.18349502	2.02E-16	1.59E-13	4.55E-15
UP_KEYWORDS	Mitosis	232	13804	256	20042	1.315782563	1.79E-16	1.59E-13	4.66E-15
INTERPRO	IPR016024:Armadillo-type fold	313	12994	346	18205	1.267406878	5.14E-18	4.04E-14	8.09E-15
GOTERM_BP_DIRECT	GO:0006468 protein phosphorylation	401	11946	447	16412	1.232468554	8.58E-19	8.70E-15	8.70E-15
KEGG_PATHWAY	hsa01100:Metabolic pathways	987	4936	1217	6891	1.132227433	4.03E-17	1.20E-14	1.20E-14
UP_KEYWORDS	SH3 domain	198	13804	215	20042	1.337096763	6.22E-16	4.78E-13	1.33E-14
UP_KEYWORDS	Helicase	136	13804	141	20042	1.400412256	1.14E-15	7.96E-13	2.15E-14
GOTERM_MF_DIRECT	GO:0044822 poly(A) RNA binding	929	11974	1120	16483	1.141812245	3.35E-17	1.17E-13	3.89E-14
GOTERM_CC_DIRECT	GO:0043231 intracellular membrane-bounded organelle	459	12629	533	17701	1.20701958	5.31E-16	7.91E-13	9.89E-14
GOTERM_BP_DIRECT	GO:0044281 small molecule metabolic process	1383	1.19E+04	1.71E+03	16412	1.113735726	2.10E-17	2.13E-13	1.06E-13
INTERPRO	IPR017441:Protein kinase, ATP binding site	339	12994	381	18205	1.246586856	1.01E-16	8.73E-13	1.25E-13
INTERPRO	IPR015943:WD40/YVTN repeat-like-containing domain	298	1.30E+04	3.30E+02	18205	1.26517367	6.33E-17	8.73E-13	1.46E-13
UP_TISSUE	Teratocarcinoma	461	13204	546	18913	1.209381134	2.06E-15	1.34E-12	2.67E-13
GOTERM_CC_DIRECT	GO:0005794 Golgi apparatus	667	12629	802	17701	1.165682575	2.46E-15	3.48E-12	3.87E-13
UP_KEYWORDS	Transit peptide	444	13804	533	20042	1.209460999	2.29E-14	1.64E-11	4.32E-13
UP_KEYWORDS	DNA repair	253	13804	287	20042	1.279896147	3.33E-14	2.39E-11	6.12E-13
UP_TISSUE	Platelet	485	13204	579	18913	1.199825483	6.82E-15	4.29E-12	7.15E-13
INTERPRO	IPR001849:Pleckstrin homology domain	256	12994	281	18205	1.276384337	1.00E-15	7.86E-12	9.82E-13
GOTERM_CC_DIRECT	GO:0005634 nucleus	3826	12629	5071	17701	1.057499557	8.43E-15	1.20E-11	1.20E-12
GOTERM_CC_DIRECT	GO:0005925 focal adhesion	343	12629	391	17701	1.229550021	1.15E-14	1.65E-11	1.50E-12
INTERPRO	IPR017986:WD40-repeat-containing domain	272	12994	301	18205	1.266048168	1.72E-15	1.40E-11	1.55E-12
UP_KEYWORDS	Microtubule	248	13804	282	20042	1.276846469	1.15E-13	8.24E-11	2.06E-12
UP_SEQ_FEATURE	repeat:WD 3	239	13613	269	20064	1.309511436	1.70E-15	3.58E-11	4.48E-12
UP_KEYWORDS	Endosome	393	13804	470	20042	1.21403386	2.57E-13	1.84E-10	4.49E-12
UP_SEQ_FEATURE	domain:SH3	164	13613	176	20064	1.37339308	2.06E-15	4.54E-11	4.54E-12
UP_SEQ_FEATURE	repeat:WD 1	246	13613	278	20064	1.3042294	1.99E-15	4.30E-11	4.78E-12
UP_SEQ_FEATURE	repeat:WD 2	246	13613	278	20064	1.3042294	1.99E-15	4.30E-11	4.78E-12
GOTERM_CC_DIRECT	GO:0005730 nucleolus	676	12629	819	17701	1.156888844	4.08E-14	5.82E-11	4.85E-12
UP_KEYWORDS	Ligase	326	13804	383	20042	1.235819186	2.90E-13	2.08E-10	4.96E-12
UP_TISSUE	Kidney	1224	13204	1572	18913	1.115279663	8.67E-14	5.49E-11	7.84E-12
UP_SEQ_FEATURE	compositionally biased region:Ser-rich	371	13613	441	20064	1.239935216	7.56E-15	1.62E-10	1.48E-11
UP_KEYWORDS	Guanine-nucleotide releasing factor	135	13804	144	20042	1.361154376	1.34E-12	9.62E-10	2.24E-11
GOTERM_CC_DIRECT	GO:0005819 spindle	111	12629	113	17701	1.376807979	3.86E-13	5.49E-10	4.23E-11
GOTERM_BP_DIRECT	GO:0010467 gene expression	796	11946	958	16412	1.141527968	1.43E-14	1.45E-10	4.84E-11
GOTERM_CC_DIRECT	GO:0005789 endoplasmic reticulum membrane	666	1.26E+04	8.11E+02	17701	1.151018261	5.02E-13	7.15E-10	5.11E-11
UP_KEYWORDS	Mental retardation	238	13804	273	20042	1.265757231	3.17E-12	2.27E-09	5.16E-11
UP_KEYWORDS	Actin-binding	233	13804	267	20042	1.267012113	4.36E-12	3.12E-09	6.94E-11
GOTERM_BP_DIRECT	GO:0016032 viral process	469	11946	544	16412	1.18443966	4.00E-14	4.05E-10	1.01E-10

UP_SEQ_FEATURE	repeat:WD 4	225	13613	255	20064	1.300486991	7.26E-14	1.56E-09	1.30E-10
UP_SEQ_FEATURE	domain:PH	217	13613	245	20064	1.305441227	8.37E-14	1.80E-09	1.39E-10
UP_KEYWORDS	Calcium	686	13804	868	20042	1.147467775	9.09E-12	6.52E-09	1.42E-10
INTERPRO	IPR001452:Src homology-3 domain	201	12994	219	18205	1.285877992	2.63E-13	2.07E-09	2.07E-10
KEGG_PATHWAY	hsa04510:Focal adhesion	189	4936	206	6891	1.280860057	1.84E-12	5.47E-10	2.74E-10

Supplementary Table 7: **Functional enrichment for isoforms with > 4 transcripts.**

Category	Term	Count	List Total	Pop Hits	Pop Total	Fold Enrichment	PValue	Bonferroni	BH FDR
UP_KEYWORDS	Alternative splicing	7362	10332	10581	20042	1.349664295	0	0	0
UP_KEYWORDS	Phosphoprotein	5277	10332	7493	20042	1.366117397	0	0	0
UP_SEQ_FEATURE	splice variant	5408	10234	7761	20064	1.366127098	0	0	0
UP_KEYWORDS	Cytoplasm	3268	10332	4750	20042	1.334581494	8.67E-167	5.98E-164	2.99E-164
UP_KEYWORDS	Acetylation	2444	10332	3426	20042	1.383791005	4.32E-147	2.97E-144	9.91E-145
UP_KEYWORDS	ATP-binding	1138	10332	1384	20042	1.595007877	1.23E-133	8.49E-131	2.12E-131
UP_KEYWORDS	Proteomics identification	1778	10332	2405	20042	1.434079858	6.40E-126	4.41E-123	8.82E-124
GOTERM_MF_DIRECT	GO:0005515 protein binding	5595	9153	8706	16483	1.157322041	7.39E-127	2.32E-123	2.32E-123
UP_KEYWORDS	Nucleotide-binding	1378	10332	1774	20042	1.50678838	3.85E-124	2.66E-121	4.43E-122
GOTERM_MF_DIRECT	GO:0005524 ATP binding	1200	9153	1467	16483	1.473071844	1.15E-108	3.60E-105	1.80E-105
GOTERM_CC_DIRECT	GO:0005829 cytosol	2272	9542	3217	17701	1.310133815	3.15E-101	4.28E-98	4.28E-98
UP_TISSUE	Epithelium	1998	10014	2816	18913	1.340033541	1.14E-98	6.53E-96	6.53E-96
UP_SEQ_FEATURE	nucleotide phosphate-binding region:ATP	820	1.02E+04	9.94E+02	20064	1.617333471	1.91E-99	3.32E-95	1.66E-95
UP_KEYWORDS	Coiled coil	1858	1.03E+04	2.65E+03	20042	1.360568499	1.97E-96	1.36E-93	1.94E-94
GOTERM_CC_DIRECT	GO:0005654 nucleoplasm	1856	9542	2561	17701	1.344394674	7.80E-96	1.06E-92	5.30E-93
GOTERM_CC_DIRECT	GO:0005737 cytoplasm	3152	9542	4799	17701	1.218411105	2.31E-83	3.14E-80	1.05E-80
UP_KEYWORDS	Polymorphism	6855	1.03E+04	1.20E+04	20042	1.105990184	9.58E-81	6.60E-78	8.25E-79
GOTERM_CC_DIRECT	GO:0016020 membrane	1407	9542	1901	17701	1.372999473	8.24E-81	1.12E-77	2.80E-78
UP_SEQ_FEATURE	sequence variant	6965	10234	12444	20064	1.097319823	6.69E-73	1.16E-68	3.87E-69
UP_TISSUE	Brain	4971	10014	8256	18913	1.137173981	3.53E-70	2.02E-67	1.01E-67
UP_KEYWORDS	Transferase	1151	10332	1627	20042	1.372285363	1.53E-60	1.05E-57	1.17E-58
UP_KEYWORDS	Kinase	572	10332	709	20042	1.564971576	4.94E-60	3.40E-57	3.40E-58
UP_KEYWORDS	Ubl conjugation	1171	1.03E+04	1.67E+03	20042	1.362630029	5.80E-59	4.00E-56	3.63E-57
UP_KEYWORDS	Cytoskeleton	819	10332	1112	20042	1.428682664	5.04E-54	3.47E-51	2.89E-52
UP_SEQ_FEATURE	mutagenesis site	1457	10234	2191	20064	1.303734868	2.00E-54	3.47E-50	8.68E-51
UP_KEYWORDS	Nucleus	3138	1.03E+04	5.18E+03	20042	1.174433392	6.30E-52	4.34E-49	3.34E-50
UP_SEQ_FEATURE	binding site:ATP	451	10234	558	20064	1.584581019	2.74E-49	4.75E-45	9.51E-46
UP_SEQ_FEATURE	active site:Proton acceptor	522	10234	672	20064	1.522906837	3.26E-47	5.66E-43	9.44E-44
UP_SEQ_FEATURE	domain:Protein kinase	394	10234	478	20064	1.615996559	4.09E-47	7.11E-43	1.02E-43
UP_KEYWORDS	Disease mutation	1591	1.03E+04	2.46E+03	20042	1.252524231	2.93E-44	2.02E-41	1.44E-42
INTERPRO	IPR011993:Pleckstrin homology-like domain	366	9861	427	18205	1.582424269	3.04E-44	2.05E-40	2.05E-40
INTERPRO	IPR011009:Protein kinase-like domain	437	9861	530	18205	1.522212528	1.53E-43	1.03E-39	5.17E-40
UP_KEYWORDS	Hydrolase	1108	10332	1653	20042	1.300240134	1.93E-40	1.33E-37	8.87E-39
INTERPRO	IPR000719:Protein kinase, catalytic domain	401	9861	486	18205	1.523273293	4.10E-40	2.77E-36	9.24E-37
UP_KEYWORDS	Serine/threonine-protein kinase	326	1.03E+04	3.95E+02	20042	1.600947774	4.44E-38	3.06E-35	1.91E-36
UP_KEYWORDS	Cell cycle	480	10332	640	20042	1.454849013	6.73E-35	4.64E-32	2.73E-33
UP_KEYWORDS	Metal-binding	2208	10332	3640	20042	1.176669092	1.03E-34	7.11E-32	3.95E-33
UP_KEYWORDS	Protein transport	457	10332	606	20042	1.462851483	3.14E-34	2.17E-31	1.14E-32
INTERPRO	IPR016024:Armadillo-type fold	296	9861	346	18205	1.579375282	1.64E-35	1.11E-31	2.77E-32
UP_KEYWORDS	WD repeat	240	1.03E+04	2.81E+02	20042	1.656767559	8.11E-33	5.59E-30	2.79E-31
INTERPRO	IPR027417:P-loop containing nucleoside triphosphate hydrolase	643	9861	871	18205	1.362895452	2.52E-34	1.70E-30	2.84E-31
INTERPRO	IPR015943:WD40/YVTN repeat-like-containing domain	283	9.86E+03	3.30E+02	18205	1.583223473	2.26E-34	1.53E-30	3.05E-31
UP_KEYWORDS	DNA damage	279	10332	341	20042	1.587108014	2.09E-31	1.44E-28	6.56E-30
UP_KEYWORDS	Cell projection	494	10332	675	20042	1.419645259	2.09E-31	1.44E-28	6.84E-30
UP_KEYWORDS	Chromatin regulator	242	10332	287	20042	1.635649064	2.84E-31	1.96E-28	8.50E-30
UP_KEYWORDS	Magnesium	414	10332	550	20042	1.460139373	9.17E-31	6.32E-28	2.63E-29

INTERPRO	IPR017986:WD40-repeat-containing domain	259	9861	301	18205	1.588557696	5.19E-32	3.51E-28	5.01E-29
INTERPRO	IPR001849:Pleckstrin homology domain	243	9861	281	18205	1.596502776	8.68E-31	5.86E-27	7.33E-28
UP_SEQ_FEATURE	repeat:WD 3	227	1.02E+04	2.69E+02	20064	1.654419665	2.54E-30	4.42E-26	5.52E-27
UP_SEQ_FEATURE	repeat:WD 2	233	10234	278	20064	1.643172779	3.36E-30	5.83E-26	6.48E-27
UP_SEQ_FEATURE	repeat:WD 1	233	10234	278	20064	1.643172779	3.36E-30	5.83E-26	6.48E-27
UP_KEYWORDS	Cell division	298	10332	378	20042	1.52925928	3.73E-28	2.57E-25	1.03E-26
INTERPRO	IPR017441:Protein kinase, ATP binding site	311	9861	381	18205	1.506971843	1.44E-29	9.72E-26	1.08E-26
UP_KEYWORDS	Isopeptide bond	687	10332	1008	20042	1.322065174	4.58E-28	3.16E-25	1.21E-26
UP_KEYWORDS	Helicase	133	10332	141	20042	1.829739184	4.97E-28	3.42E-25	1.27E-26
UP_KEYWORDS	DNA repair	236	10332	287	20042	1.595095782	2.74E-27	1.89E-24	6.75E-26
GOTERM_BP_DIRECT	GO:0006468 protein phosphorylation	362	9148	447	16412	1.452902261	9.27E-30	8.51E-26	8.51E-26
GOTERM_CC_DIRECT	GO:0005813 centrosome	331	9542	416	17701	1.476022756	4.94E-28	6.72E-25	1.34E-25
INTERPRO	IPR001680:WD40 repeat	231	9861	271	18205	1.573665463	2.32E-27	1.57E-23	1.57E-24
UP_SEQ_FEATURE	repeat:WD 4	213	10234	255	20064	1.637613951	2.90E-27	5.04E-23	5.04E-24
GOTERM_MF_DIRECT	GO:0044822 poly(A) RNA binding	791	9153	1120	16483	1.27183642	5.32E-27	1.67E-23	5.57E-24
UP_TISSUE	Platelet	428	10014	579	18913	1.396104866	9.01E-26	5.15E-23	1.72E-23
UP_KEYWORDS	SH3 domain	183	10332	215	20042	1.651084461	8.38E-25	5.78E-22	1.99E-23
UP_TISSUE	Placenta	2159	10014	3558	18913	1.146039362	1.97E-25	1.12E-22	2.25E-23
UP_TISSUE	Uterus	1179	10014	1833	18913	1.2147983	1.81E-25	1.03E-22	2.58E-23
UP_KEYWORDS	Host-virus interaction	295	10332	384	20042	1.490209926	1.42E-24	9.75E-22	3.25E-23
UP_SEQ_FEATURE	domain:PH	204	1.02E+04	2.45E+02	20064	1.632436097	8.70E-26	1.51E-21	1.37E-22
UP_SEQ_FEATURE	repeat:WD 5	198	10234	237	20064	1.637905913	2.05E-25	3.57E-21	2.97E-22
UP_KEYWORDS	Ligase	291	10332	383	20042	1.47384182	5.97E-23	4.11E-20	1.33E-21
SMART	SM00233:SM00233	243	5556	281	9460	1.472410321	3.00E-24	2.08E-21	2.08E-21
GOTERM_MF_DIRECT	GO:0004674 protein serine/threonine kinase activity	271	9153	331	16483	1.474395828	3.86E-24	1.21E-20	3.03E-21
INTERPRO	IPR008271:Serine/threonine-protein kinase, active site	254	9861	312	18205	1.50296493	5.77E-24	3.89E-20	3.54E-21
GOTERM_BP_DIRECT	GO:0000278 mitotic cell cycle	338	9148	428	16412	1.416799137	3.57E-24	3.28E-20	1.64E-20
UP_KEYWORDS	Mental retardation	217	10332	273	20042	1.541891261	1.48E-21	1.02E-18	3.19E-20
GOTERM_CC_DIRECT	GO:0043231 intracellular membrane-bounded organelle	395	9542	533	17701	1.374764397	2.32E-22	3.15E-19	5.25E-20
UP_KEYWORDS	Guanine-nucleotide releasing factor	128	10332	144	20042	1.724265497	4.95E-21	3.41E-18	1.03E-19
INTERPRO	IPR011989:Armadillo-like helical	182	9861	212	18205	1.584912357	1.93E-22	1.30E-18	1.08E-19
UP_SEQ_FEATURE	domain:SH3	152	10234	176	20064	1.693179597	1.28E-22	2.23E-18	1.71E-19
UP_TISSUE	Testis	2645	10014	4482	18913	1.11456823	2.03E-21	1.16E-18	1.94E-19
UP_KEYWORDS	Mitosis	204	10332	256	20042	1.545777076	1.48E-20	1.02E-17	2.99E-19
GOTERM_CC_DIRECT	GO:0005925 focal adhesion	301	9542	391	17701	1.428065502	1.94E-21	2.64E-18	3.30E-19
GOTERM_CC_DIRECT	GO:0005794 Golgi apparatus	561	9542	802	17701	1.297618064	1.74E-21	2.36E-18	3.38E-19
GOTERM_CC_DIRECT	GO:0005730 nucleolus	571	9542	819	17701	1.293333707	2.41E-21	3.28E-18	3.65E-19
INTERPRO	IPR001452:Src homology-3 domain	186	9861	219	18205	1.567972906	8.57E-22	5.79E-18	4.45E-19
GOTERM_CC_DIRECT	GO:0005739 mitochondrion	824	9542	1238	17701	1.234709975	3.71E-21	5.05E-18	5.05E-19
UP_KEYWORDS	Transport	1202	10332	1965	20042	1.186584233	5.90E-20	4.06E-17	1.16E-18
SMART	SM00320:SM00320	230	5556	270	9460	1.4504173	3.91E-21	2.70E-18	1.35E-18
INTERPRO	IPR002290:Serine/threonine- / dual specificity protein kinase, catalytic domain	223	9861	274	18205	1.502533019	4.08E-21	2.75E-17	1.97E-18
UP_SEQ_FEATURE	compositionally biased region:Ser-rich	322	10234	441	20064	1.431493528	1.95E-21	3.39E-17	2.42E-18
UP_KEYWORDS	Golgi apparatus	539	10332	806	20042	1.297210286	1.74E-19	1.20E-16	3.33E-18
UP_KEYWORDS	Endoplasmic reticulum	685	10332	1057	20042	1.257106999	2.12E-19	1.46E-16	3.96E-18

GOTERM_BP_DIRECT	GO:0006281 DNA repair	300	9148	380	16412	1.416357904	1.63E-21	1.50E-17	4.98E-18
INTERPRO	IPR014001:Helicase, superfamily 1/2, ATP-binding domain	104	9861	110	18205	1.745461921	4.08E-20	2.76E-16	1.84E-17
UP_KEYWORDS	GTPase activation	160	10332	195	20042	1.591629689	1.37E-18	9.46E-16	2.49E-17
INTERPRO	IPR001650:Helicase, C-terminal	102	9861	108	18205	1.743597111	1.23E-19	8.31E-16	5.19E-17
UP_SEQ_FEATURE	repeat:WD 6	162	10234	196	20064	1.620432891	5.35E-20	9.29E-16	6.19E-17
GOTERM_BP_DIRECT	GO:0043547 positive regulation of GTPase activity	364	9.15E+03	4.81E+02	16412	1.357661991	3.94E-20	3.62E-16	9.04E-17
UP_TISSUE	T-cell	256	10014	338	18913	1.43046126	4.52E-18	2.59E-15	3.69E-16
UP_SEQ_FEATURE	domain:Helicase C-terminal	99	10234	108	20064	1.797146766	5.68E-19	9.86E-15	6.16E-16
UP_KEYWORDS	Endosome	331	10332	470	20042	1.366113541	3.61E-17	2.49E-14	6.38E-16

Supplementary Table 8: **Functional enrichment for isoforms with > 6 transcripts.**

Category	Term	Count	List Total	Pop Hits	Pop Total	Fold Enrichment	PValue	Bonferroni	BH FDR
UP_KEYWORDS	Alternative splicing	4639	6096	10581	20042	1.441430667	0	0	0
UP_KEYWORDS	Phosphoprotein	3462	6096	7493	20042	1.519033775	3.70E-303	2.35E-300	1.18E-300
UP_SEQ_FEATURE	splice variant	3513	6055	7761	20064	1.499905294	3.68E-295	4.33E-291	4.33E-291
UP_KEYWORDS	ATP-binding	859	6096	1384	20042	2.040577873	3.03E-140	1.93E-137	6.43E-138
UP_KEYWORDS	Cytoplasm	2143	6096	4750	20042	1.483285191	2.72E-134	1.73E-131	4.32E-132
UP_TISSUE	Epithelium	1430	5944	2816	18913	1.615790345	1.63E-119	7.86E-117	7.86E-117
GOTERM_MF_DIRECT	GO:0005524 ATP binding	899	5500	1467	16483	1.836551651	2.35E-116	5.76E-113	5.76E-113
UP_KEYWORDS	Nucleotide-binding	978	6096	1774	20042	1.812513871	7.46E-114	4.75E-111	9.49E-112
UP_SEQ_FEATURE	nucleotide phosphate-binding region:ATP	632	6055	994	20064	2.106852178	6.42E-110	7.54E-106	3.77E-106
UP_KEYWORDS	Polymorphism	4308	6096	12023	20042	1.178037043	4.08E-95	2.60E-92	4.33E-93
UP_KEYWORDS	Coiled coil	1272	6096	2649	20042	1.578705974	4.73E-93	3.01E-90	4.30E-91
UP_SEQ_FEATURE	sequence variant	4367	6055	12444	20064	1.162857668	2.79E-86	3.28E-82	1.09E-82
UP_KEYWORDS	Acetylation	1526	6096	3426	20042	1.464411985	1.39E-82	8.87E-80	1.11E-80
GOTERM_MF_DIRECT	GO:0005515 protein binding	3484	5500	8706	16483	1.199314412	5.84E-83	1.43E-79	7.15E-80
GOTERM_CC_DIRECT	GO:0005654 nucleoplasm	1239	5703	2561	17701	1.501606565	5.42E-76	6.56E-73	6.56E-73
GOTERM_CC_DIRECT	GO:0005829 cytosol	1475	5703	3217	17701	1.423099906	7.38E-72	8.93E-69	4.47E-69
UP_TISSUE	Brain	3137	5944	8256	18913	1.209000432	6.27E-66	3.03E-63	1.51E-63
UP_KEYWORDS	Cytoskeleton	597	6096	1112	20042	1.765085078	7.87E-62	5.01E-59	5.56E-60
GOTERM_CC_DIRECT	GO:0016020 membrane	942	5703	1901	17701	1.538024368	3.90E-62	4.72E-59	1.57E-59
UP_KEYWORDS	Proteomics identification	1086	6096	2405	20042	1.484604744	1.28E-59	8.13E-57	8.13E-58
GOTERM_CC_DIRECT	GO:0005737 cytoplasm	1999	5703	4799	17701	1.292874812	3.06E-59	3.71E-56	9.27E-57
UP_KEYWORDS	Kinase	414	6096	709	20042	1.919774441	3.28E-55	2.09E-52	1.90E-53
INTERPRO	IPR016024:Armadillo-type fold	253	5863	346	18205	2.270467091	3.91E-55	1.92E-51	1.92E-51
UP_KEYWORDS	Ubl conjugation	792	6096	1667	20042	1.562016731	6.11E-53	3.89E-50	3.24E-51
INTERPRO	IPR011993:Pleckstrin homology-like domain	290	5863	427	18205	2.108826799	3.19E-52	1.56E-48	7.81E-49
UP_SEQ_FEATURE	binding site:ATP	333	6055	558	20064	1.977485949	4.82E-48	5.67E-44	1.42E-44
INTERPRO	IPR011009:Protein kinase-like domain	331	5863	530	18205	1.939201388	3.28E-47	1.61E-43	5.36E-44
UP_KEYWORDS	Transferase	754	6096	1627	20042	1.523631323	3.14E-45	2.00E-42	1.54E-43
UP_SEQ_FEATURE	mutagenesis site	957	6055	2191	20064	1.447347889	8.06E-46	9.48E-42	1.90E-42
UP_KEYWORDS	Disease mutation	1053	6096	2464	20042	1.405024079	8.55E-44	5.44E-41	3.89E-42
UP_SEQ_FEATURE	domain:Protein kinase	291	6055	478	20064	2.017290596	2.26E-44	2.65E-40	4.42E-41
INTERPRO	IPR015943:WD40/YVTN repeat-like-containing domain	229	5863	330	18205	2.154727386	1.13E-43	5.56E-40	1.39E-40
UP_KEYWORDS	Serine/threonine-protein kinase	249	6096	395	20042	2.07251819	4.46E-41	2.84E-38	1.89E-39
INTERPRO	IPR000719:Protein kinase, catalytic domain	301	5863	486	18205	1.923096225	6.65E-42	3.26E-38	6.52E-39
UP_SEQ_FEATURE	active site:Proton acceptor	367	6055	672	20064	1.809673233	1.04E-40	1.22E-36	1.75E-37
INTERPRO	IPR017986:WD40-repeat-containing domain	209	5863	301	18205	2.156009051	5.96E-40	2.92E-36	4.87E-37
UP_KEYWORDS	Helicase	118	6096	141	20042	2.751433331	2.20E-38	1.40E-35	8.73E-37
UP_KEYWORDS	WD repeat	190	6096	281	20042	2.223020054	1.33E-37	8.48E-35	4.99E-36
UP_TISSUE	Testis	1761	5944	4482	18913	1.250170151	4.52E-38	2.18E-35	7.28E-36
INTERPRO	IPR017441:Protein kinase, ATP binding site	244	5863	381	18205	1.988545991	1.86E-37	9.13E-34	1.30E-34
INTERPRO	IPR027417:P-loop containing nucleoside triphosphate hydrolase	458	5863	871	18205	1.632744059	4.01E-37	1.97E-33	2.46E-34
UP_KEYWORDS	Hydrolase	730	6096	1653	20042	1.451931428	4.71E-35	3.00E-32	1.66E-33
INTERPRO	IPR011989:Armadillo-like helical	156	5863	212	18205	2.284859641	1.18E-34	5.79E-31	6.43E-32
INTERPRO	IPR001849:Pleckstrin homology domain	191	5863	281	18205	2.110560648	1.40E-34	6.85E-31	6.85E-32

UP_SEQ_FEATURE	repeat:WD 1	184	6055	278	20064	2.193190716	4.78E-35	5.62E-31	7.03E-32
UP_SEQ_FEATURE	repeat:WD 2	184	6055	278	20064	2.193190716	4.78E-35	5.62E-31	7.03E-32
UP_KEYWORDS	Cell projection	351	6096	675	20042	1.709619423	1.84E-32	1.17E-29	6.15E-31
UP_SEQ_FEATURE	repeat:WD 3	178	6055	269	20064	2.19265899	6.64E-34	7.80E-30	8.67E-31
INTERPRO	IPR001680:WD40 repeat	184	5863	271	18205	2.108236467	3.06E-33	1.50E-29	1.37E-30
UP_KEYWORDS	Nucleus	1911	6096	5183	20042	1.212203623	1.09E-31	6.91E-29	3.46E-30
UP_SEQ_FEATURE	domain:PH	163	6055	245	20064	2.204575069	1.60E-31	1.88E-27	1.88E-28
UP_SEQ_FEATURE	repeat:WD 5	158	6055	237	20064	2.209083402	9.92E-31	1.17E-26	1.06E-27
UP_SEQ_FEATURE	repeat:WD 4	166	6055	255	20064	2.157104969	2.08E-30	2.45E-26	2.04E-27
GOTERM_MF_DIRECT	GO:0004674 protein serine/threonine kinase activity	212	5500	331	16483	1.919470475	2.76E-30	6.76E-27	2.25E-27
INTERPRO	IPR001650:Helicase, C-terminal	92	5863	108	18205	2.645055938	4.11E-29	2.02E-25	1.68E-26
INTERPRO	IPR014001:Helicase, superfamily 1/2, ATP-binding domain	93	5863	110	18205	2.625191881	6.47E-29	3.17E-25	2.44E-26
UP_KEYWORDS	Chromatin regulator	177	6096	287	20042	2.027624215	9.68E-28	6.15E-25	2.93E-26
UP_KEYWORDS	Guanine-nucleotide releasing factor	108	6096	144	20042	2.465797244	1.49E-27	9.45E-25	4.29E-26
GOTERM_CC_DIRECT	GO:0005813 centrosome	243	5703	416	17701	1.813039949	1.99E-28	2.41E-25	4.82E-26
UP_SEQ_FEATURE	domain:Helicase ATP-binding	93	6055	114	20064	2.703220479	6.09E-29	7.16E-25	5.51E-26
UP_SEQ_FEATURE	domain:Helicase C-terminal	89	6055	108	20064	2.730672539	2.50E-28	2.94E-24	2.10E-25
UP_KEYWORDS	DNA damage	199	6096	341	20042	1.918645754	1.05E-26	6.69E-24	2.91E-25
INTERPRO	IPR002290:Serine/threonine- / dual specificity protein kinase, catalytic domain	176	5863	274	18205	1.994494734	1.98E-27	9.71E-24	6.94E-25
UP_KEYWORDS	Isopeptide bond	462	6096	1008	20042	1.506876094	3.27E-26	2.08E-23	8.65E-25
UP_KEYWORDS	Cell cycle	319	6096	640	20042	1.638727752	1.80E-25	1.15E-22	4.59E-24
UP_KEYWORDS	SH3 domain	139	6096	215	20042	2.125555454	8.49E-25	5.40E-22	2.08E-23
INTERPRO	IPR008271:Serine/threonine-protein kinase, active site	191	5863	312	18205	1.900857507	6.36E-26	3.12E-22	2.08E-23
GOTERM_BP_DIRECT	GO:0043547 positive regulation of GTPase activity	275	5487	481	16412	1.710071092	3.74E-27	2.86E-23	2.86E-23
UP_SEQ_FEATURE	compositionally biased region:Ser-rich	239	6055	441	20064	1.7958195	4.41E-26	5.19E-22	3.46E-23
INTERPRO	IPR011011:Zinc finger, FYVE/PHD-type	105	5863	138	18205	2.362549963	1.75E-25	8.58E-22	5.36E-23
UP_SEQ_FEATURE	repeat:WD 6	131	6055	196	20064	2.214718819	9.09E-26	1.07E-21	6.68E-23
UP_KEYWORDS	DNA repair	167	6096	287	20042	1.913069174	2.23E-22	1.42E-19	5.25E-21
INTERPRO	IPR003593:AAA+ ATPase domain	106	5863	145	18205	2.269910073	2.24E-23	1.10E-19	6.45E-21
UP_KEYWORDS	Ligase	206	6096	383	20042	1.768335012	1.21E-21	7.67E-19	2.65E-20
UP_KEYWORDS	Protein transport	295	6096	606	20042	1.600462458	1.19E-21	7.54E-19	2.69E-20
UP_TISSUE	Placenta	1358	5944	3558	18913	1.21443828	5.94E-22	2.87E-19	7.18E-20
INTERPRO	IPR001452:Src homology-3 domain	141	5863	219	18205	1.999151867	2.75E-22	1.35E-18	7.48E-20
GOTERM_BP_DIRECT	GO:0006468 protein phosphorylation	251	5487	447	16412	1.679549262	2.36E-23	1.81E-19	9.03E-20
UP_KEYWORDS	Mental retardation	158	6096	273	20042	1.902788594	6.58E-21	4.18E-18	1.39E-19
GOTERM_CC_DIRECT	GO:0030027 lamellipodium	109	5703	157	17701	2.154870998	3.74E-21	4.52E-18	7.53E-19
UP_SEQ_FEATURE	domain:SH3	115	6055	176	20064	2.165152766	1.62E-21	1.91E-17	1.12E-18
UP_KEYWORDS	GTPase activation	121	6096	195	20042	2.040078404	1.45E-19	9.20E-17	2.97E-18
UP_KEYWORDS	Cell division	198	6096	378	20042	1.722144107	3.68E-19	2.34E-16	7.32E-18
UP_TISSUE	Teratocarcinoma	272	5944	546	18913	1.585104442	1.14E-19	5.50E-17	1.10E-17
UP_KEYWORDS	Actin-binding	151	6096	267	20042	1.859352728	9.31E-19	5.92E-16	1.79E-17
UP_SEQ_FEATURE	repeat:WD 7	101	6055	151	20064	2.216398248	4.28E-20	5.04E-16	2.80E-17
GOTERM_BP_DIRECT	GO:0046777 protein autophosphorylation	115	5487	167	16412	2.059718725	2.10E-20	1.60E-16	5.35E-17
GOTERM_MF_DIRECT	GO:0005096 GTPase activator activity	159	5500	263	16483	1.811819564	2.55E-19	6.25E-16	1.56E-16

GOTERM_BP_DIRECT	GO:0000278 mitotic cell cycle	233	5487	428	16412	1.628316037	1.78E-19	1.36E-15	3.40E-16
GOTERM_MF_DIRECT	GO:0044822 poly(A) RNA binding	507	5500	1120	16483	1.356636526	4.98E-18	1.22E-14	2.44E-15
INTERPRO	IPR001965:Zinc finger, PHD-type	70	5863	91	18205	2.388512051	1.23E-17	6.05E-14	3.18E-15
UP_KEYWORDS	Magnesium	258	6096	550	20042	1.542244094	2.60E-16	1.41E-13	4.11E-15
UP_KEYWORDS	Transport	758	6096	1965	20042	1.268243807	3.57E-16	2.12E-13	6.11E-15
UP_SEQ_FEATURE	compositionally biased region:Poly-Glu	253	6055	531	20064	1.578808194	9.95E-18	1.17E-13	6.15E-15
UP_TISSUE	Uterus	735	5944	1833	18913	1.275870206	1.57E-16	5.36E-14	8.88E-15
UP_KEYWORDS	Metal-binding	1311	6096	3640	20042	1.184124611	7.66E-16	4.94E-13	1.38E-14
UP_TISSUE	Platelet	276	5944	579	18913	1.516743492	2.05E-16	1.07E-13	1.53E-14
INTERPRO	IPR019775:WD40 repeat, conserved site	106	5863	166	18205	1.982752775	1.26E-16	5.44E-13	2.72E-14
GOTERM_CC_DIRECT	GO:0005794 Golgi apparatus	368	5703	802	17701	1.424189832	1.97E-16	2.69E-13	3.84E-14
UP_KEYWORDS	TPR repeat	101	6096	167	20042	1.988387398	2.67E-15	1.69E-12	4.59E-14

Supplementary Table 9: **Model and variational parameters, conditional distributions and expectations.** Model parameters (arguments of posterior distribution density), their variational distribution and counterpart variational parameters, the complete conditional parameter and relevant expectations.

Model param.	Distribution	Variational param.	Conditional distribution	Relevant expectation(s)
z_{ij}	multinomial (*)	ϕ_{ij}	$\log(\psi_{jl}) + \sum_{k=1}^{K} \mathbb{I}_{c_{jl}=k} \log(\beta_{k,w_{ij}})$	$\mathbb{E}(\mathbb{I}_{z_{ji}=c_{jl}}) = \phi_{ij}^{l}$
$\sigma_{jl}^{-1}(\psi)$	beta	$\left(\gamma_{jl}^{1}, \gamma_{jl}^{2}\right)$	$\left(1 + \sum_{i=1}^{N_j} \mathbb{I}_{z_{ji}=c_{jl}}, \alpha + \sum_{i=1}^{N_j} \sum_{k>l} \mathbb{I}_{z_{ji}=c_{jk}}\right)$	$\mathbb{E}(\log(\psi_{jl})) = \boldsymbol{\psi}(\gamma_{jl}^{1}) - \boldsymbol{\psi}(\gamma_{jl}^{1} + \gamma_{jl}^{2}) + \sum_{x=1}^{l-1} \left(\boldsymbol{\psi}(\gamma_{jx}^{2}) - \boldsymbol{\psi}(\gamma_{jx}^{1} + \gamma_{jx}^{2})\right)$
c_{jl}	multinomial (*)	ζ_{jl}	$\log(t_k) + \sum_{i=1}^{N_j} \mathbb{I}_{z_{ji}=c_{jl}} \log(\beta_{k,w_{ji}})$	$\mathbb{E}(\mathbb{I}_{c_{jl}=k}) = \zeta_{jl}^{k}$
$\sigma_{k}^{-1}(t)$	beta	$\left(a_{k}^{1}, a_{k}^{2}\right)$	$\left(1 + \sum_{j=1}^{M} \sum_{l=1}^{T} \mathbb{I}_{c_{jl}=k}, \omega + \sum_{j=1}^{M} \sum_{l=1}^{T} \sum_{x>l} \mathbb{I}_{c_{jl}=x}\right)$	$\mathbb{E}(\log(t_k)) = \boldsymbol{\psi}(a_{k}^{1}) - \boldsymbol{\psi}(a_{k}^{1} + a_{k}^{2}) + \sum_{x=1}^{k-1} \left(\boldsymbol{\psi}(a_{x}^{2}) - \boldsymbol{\psi}(a_{x}^{1} + a_{x}^{2})\right)$
β_k	dirichlet	λ_k	$\left(\eta + \sum_{j=1}^{M} \sum_{l=1}^{T} \mathbb{I}_{c_{jl}=k} \sum_{i=1}^{N_j} \mathbb{I}_{z_{ji}=c_{jl}} \mathbb{I}_{w_{ji}=v}\right)$	$\mathbb{E}(\log(\beta_{kv})) = \boldsymbol{\psi}(\lambda_{kv}) - \boldsymbol{\psi}\left(\sum_{v'=1}^{V} \lambda_{kv'}\right)$
π_k	beta	$\left(\rho_{k}^{1}, \rho_{k}^{2}\right)$	$\left(r + \sum_{e=1}^{E} b'_{ke}, s + E - \sum_{e=1}^{E} b'_{ke}\right)$	$\mathbb{E}(\log \pi_k) = \boldsymbol{\psi}(\rho_{k}^{1}) - \boldsymbol{\psi}(\rho_{k}^{1} + \rho_{k}^{2})$
				$\mathbb{E}(\log(1 - \pi_k)) = \boldsymbol{\psi}(\rho_{k}^{2}) - \boldsymbol{\psi}(\rho_{k}^{1} + \rho_{k}^{2})$
b'_{ke}	bernoulli	μ_{ke}	$\log P(b'_{ke} = 1 \| \pi_k) + \log P(\beta_k \| b'_{ke} = 1, (b'_{kf})_{f \neq e}, \eta, exons)$	$\mathbb{E}(b'_{ke}) = \mu_{ke}$, $\mathbb{E}(b'_{kv})$

Supplementary Table 10: **Updates for variational parameters in the BIISQ model.**

$$\phi^l_{ji} \propto \exp(E_q \log \psi_{jl} + \sum_{k=1}^{K} E_q \mathbb{I}(c_{jl} = k) E_q \log \beta_{k,w_{ji}})$$

$$\phi^l_{ji} \propto \exp\left(\boldsymbol{\psi}(\gamma^1_{jl}) - \boldsymbol{\psi}(\gamma^1_{jl} + \gamma^2_{jl}) + \sum_{x=1}^{l-1} \boldsymbol{\psi}(\gamma^2_{jx}) - \boldsymbol{\psi}(\gamma^1_{jx} + \gamma^2_{jx})\right)$$

$$\prod_{k=1}^{K} \exp \zeta^k_{jl} * \exp\left(\boldsymbol{\psi}(\lambda_{kv}) - \boldsymbol{\psi}(\sum_{v'=1}^{V} \lambda_{kv'})\right)$$

$$\gamma^1_{jl} \leftarrow 1 + \sum_{i=1}^{N_j} E_q \mathbb{I}(z_{ji} = c_{jl}) \leftarrow 1 + \sum_{i=1}^{N_j} \phi^l_{ji}$$

$$\gamma^2_{jl} \leftarrow \alpha + \sum_{i=1}^{N_j} \sum_{x>l} E_q \mathbb{I}(z_{ji} = c_{jx}) \leftarrow \alpha + \sum_{i=1}^{N_j} \sum_{x>l} \phi^x_{ji}$$

$$\zeta^k_{jl} \propto \exp\left(E_q \log t_k + \sum_{i=1}^{N_j} E_q \mathbb{I}(z_{ji} = c_{jl}) E_q \log \beta_{k,w_{ji}}\right)$$

$$\zeta^k_{jl} \propto \exp\left(\boldsymbol{\psi}(a^1_{jl}) - \boldsymbol{\psi}(a^1_{jl} + a^2_{jl}) + \sum_{x=1}^{l-1} \boldsymbol{\psi}(a^2_{jx}) - \boldsymbol{\psi}(a^1_{jx} + a^2_{jx})\right)$$

$$\prod_{i=1}^{N_j} \exp \phi^l_{ji} * \exp\left(\boldsymbol{\psi}(\lambda_{kv}) - \boldsymbol{\psi}(\sum_{v'=1}^{V} \lambda_{kv'})\right)$$

$$a^1_k \leftarrow 1 + \sum_{j=1}^{M} \sum_{l=1}^{T} E_q \mathbb{I}(c_{jl} = k) \leftarrow 1 + \sum_{j=1}^{M} \sum_{l=1}^{T} \zeta^k_{jl}$$

$$a^2_k \leftarrow \omega + \sum_{j=1}^{M} \sum_{l=1}^{T} \sum_{x>k} E_q \mathbb{I}(c_{jl} = x) \leftarrow \omega + \sum_{j=1}^{M} \sum_{l=1}^{T} \sum_{x>k} \zeta^x_{jl}$$

$$\lambda^v_k \leftarrow E_q b_{kv} * \left(\eta + \sum_{j=1}^{M} \sum_{l=1}^{T} E_q \mathbb{I}_{c_{jl}=k} \sum_{i=1}^{N_j} E_q \mathbb{I}_{z_{ji}=c_{jl}} * \mathbb{I}_{w_{ji}=v}\right)$$

$$\lambda^v_k \leftarrow E_q b_{kv} * \left(\eta + \sum_{j=1}^{M} \sum_{l=1}^{T} \zeta j l^k \sum_{i=1}^{N_j} \phi^l_{ji} * \mathbb{I}_{w_{ji}=v}\right)$$

Supplementary Table 11: **PEER factor optimization.**

correction	peer K	var-gene pairs	genes
none	5	23462	2060
FDR	5	319	33
none	10	22955	2094
FDR	10	310	35
none	15	23384	2095
FDR	15	314	35
none	20	22855	2107
FDR	20	272	26
none	25	22722	2093
FDR	25	269	29
none	30	23361	2092
FDR	30	267	25
none	35	22842	2091
FDR	35	267	26
none	40	23036	2079
FDR	40	266	22
none	45	22535	2061
FDR	45	283	28
none	50	22852	2090
FDR	50	218	17

Methods

BEERS simulations. We used the *benchmarker for evaluating the effectiveness of RNA-Seq software* (BEERS)[4] software to generate isoforms and reads for the initial RNA-seq simulations. In total, we simulated $3,102$ genes and $532,800$ RNA-seq samples by varying the following parameters: number of novel transcripts in {2,4,6,12,16}, the minimum gene coverage in {1,5,15,50,100}, the number of samples in {100,250}, and read lengths in {200,400} (for 30 genes). BEERS combinatorially defines splice forms by preferentially retaining or removing exons for each sampled gene model until the number of novel transcripts defines in the program input is generated. We did not simulate reads with sequencing or read mapping errors, intron contamination, substitutions or indels, or low quality read tails. The output of this simulation is a file with a row for each read with entries being the sample, mapped isoform, covered exons, start position, reference strand, sequence read content, and CIGAR mapping string; this file was used to generate the input for BIISQ, CEM, Cufflinks, and SLIDE.

PacBio simulations We downloaded full length non-chimeric human transcriptome data from liver, heart, and brain from the Iso-Seq protocol which included unaligned sequence read and General Feature Format (GFF) reference files for each tissue[1]. We constructed a reference set of genes and their transcripts across tissues. For each gene, we created a standard set of exons by parsing its transcripts and collapsing overlapping exons. We then mapped genes across the three tissues based on a base-pair overlap of 95%; genes without unique mappings were discarded. For each gene, we then mapped transcripts across tissues: transcripts were successfully mapped if the mapped transcript contained the same number of exons as the original, and the positions of their exons overlapped by 95%. This process was conservative by design to develop a confident baseline of trans-tissue isoforms and yielded seven genes having at least two isoform transcripts across two or more tissues (see Supplementary Table 1): *BLOC1S6*, *ZFAND6*, *CYTH1*, *APP*, *C1orf43*, *SPARCL1*, and *RNF14*.

We extracted the gene sequences of the identified transcripts from human genome version hg19 (Supplementary Table 2). We mapped the Iso-Seq sequence reads to the gene sequences

using both GMAP and STARlong algorithms[5]. We constructed an Iso-Seq short-read simulator (ISSRS) that includes sequencing parameters, a gene reference file containing exon boundaries, and an aligned input file. ISSRS produces SAM files that contain shorter sequence reads but retain the biases and errors present in the Iso-Seq data by copying the CIGAR substring of the shorter sequence reads. In brief, the simulator works as follows: (1) compute usable Iso-Seq reads: for each mapped Iso-Seq read, determine if the proportion t of its mapped bases align to exons from the reference file; (2) for each usable aligned read, determine the amount of sampling based on an input coverage c; (3) sample reads from each usable aligned read by attempting to add insert sizes distributed normally with mean 10bp and 40bp standard deviation; (4) output sampled reads while preserving the CIGAR string from the aligned Iso-Seq transcripts in SAM and BIISQ format. For step (1), STARlong mappings yielded fewer false positives than GMAP but GMAP produced many more usable alignments. Because aligned Iso-Seq transcripts are not guaranteed to completely overlap with exons, step (3) is repeated until 1000 failures in which case the transcript is skipped. This can occur when the sequencing parameters and the Iso-Seq transcript do not produce useful reads.

For the seven transcripts defined in Supplementary Table 2, we simulated reads with lengths 50bp, 100bp, and 200bp and approximate coverage values (of the input Iso-Seq transcripts) of 0.25, 0.5, 1, and 2 for 50 replicates of the brain and heart tissues (liver did not express these transcripts). We discarded simulations where an exon was not sampled by any individual and simulated reads that did not match the exons of an expressed isoform. The coverage values were approximate due to removing Iso-Seq transcripts that did not generate useful read alignments.

GEUVADIS data processing The Genetic European Variation in Health and Disease (GEUVADIS) RNA-sequencing project consists of RNA-seq data from lymphoblastoid cell lines (LCLs) of 666 individuals from the 1000 Genomes Project[6]. BIISQ requires read-terms – mapped RNA-seq read start positions, end positions, and exons covered tuples – and a model of transcription for each gene indicating contiguous transcribed subsequences including exons or retained introns.

To build the transcription model, we first extracted the protein coding and basic (subset of

representative transcripts for each gene) transcripts from the comprehensive gene annotations in GENCODE release 19 for human genome assembly version GRCh37.p13. We then built a set of representative exons for each protein coding gene. Most genes had a single transcript annotated as *basic*; for the remaining genes, we retained the transcript with the largest number of exons.

To build the read-terms, we mapped the raw RNA-seq reads with STAR 2-pass to the human genome version 19. Then, we filtered unmapped, non-primary reads that failed quality checks or were marked as duplicates and intersected the remaining mapped reads to our model of transcription (producing the intersection file). The full catalog of read-terms is built by parsing the intersection files of each sample. Each intersection file is then parsed again with the read-term catalog to determine which read-terms are expressed by each sample. A final step reduces the number of read-terms by collapsing read-terms with a similar start position and exon content to an approximate target number of read-terms of 2500.

Execution of isoform quantification methods We compared results from BIISQ with the deterministic method Cufflinks[7], CEM[8], SLIDE[9], and ISP[10]. We describe below parameter settings for each method.

Parameter settings for each method. To run BIISQ, we initialized $K = 1$, allowing the global distribution of isoforms for a given gene to consist of a single isoform expressing all exons. For the BEERS simulations, we set BIISQ hyperparameters to maximize precision and recall on a random held out RefSeq gene. The hyperparameters were as follows: $N - iter = 5000$, $threads = 1$, $use - cython = 1$, $max - n - prop = 3$, $min - n - prop = 3$, $iter - prop = 30$, $new - iso - prop = 2$, $red - iso - prop = 0$, $r = 0.7$, $s = 1$, $converge = 1e - 3$, $\alpha \in \{15, 100\}$, $\omega \in \{8, 50\}$, and $\eta \in \{8, 50\}$. Solutions yielding the maximum likelihood of the data were considered for evaluation. Hyperparameters for PacBio runs were the same as BEERS except $r = 1.1$. For GEUVADIS, hyperparameters were set to the same as PacBio with exceptions: $alpha = 15$, $omega = 8$, $eta = 8$.

We ran related methods as follows:

- **GMAP v.2015-12-31** [11]: `-D [data dir] -d pacbio -f samse -n 0 -t 16 --nofails [input fasta] > [output SAM] 2> [log file]`
- **starLONG v.020201** [12]: `--genomeDir [genome reference] --runThreadN 1 --readFilesIn [input fasta] --outFileNamePrefix [output] --runMode alignReads --outSAMattributes NH HI NM MD --readNameSeparator space --outFilterMultimapScoreRange 1 --outFilterMismatchNmax 2000 --scoreGapNoncan -20 --scoreGapGCAG -4 --scoreGapATAC -8 --scoreDelOpen -1 --scoreDelBase -1 --scoreInsOpen -1 --scoreInsBase -1 --alignEndsType Local --seedSearchStartLmax 50 --seedPerReadNmax 100000 --seedPerWindowNmax 1000 --alignTranscriptsPerReadNmax 100000 --alignTranscriptsPerWindowNmax 10000`
- **CEM v.2.6** [13]: `python runcem.py [input BAM]`
- **Cufflinks v.2.2.1** [7]: `cufflinks --library-type ff-firststrand -g [GTF reference] -o [output] [input BAM]`
- **SLIDE v.2012-02-17 (modification date)**[9]: `python slide.py [GTF reference] [BAM input] [GTF output] --read_type mixed`
- **ISP v.0.3** [10]: `runminst -t [output] [BAM input]`

We note that CEM was more accurate when run without reference gene annotations. We rescaled inferred RPKM in the simulated data for each gene by the ratio of the number of reads aligning to the gene to the average number of fragments in the GEUVADIS experimental data ($76,753,454$).

Statistics for evaluation. Isoform discovery results are evaluated under two independent sets of assumptions: perfect reconstruction and maximum cardinality minimum weight matchings[14]. A perfect reconstruction is analogous to conventional precision and recall (see Supplementary Figure

13). Precision and recall were calculated based exact full length isoform matches between true (simulated) and estimated isoforms in the usual way (i.e., precision = true positives (TP)/(TP+ false positives (FP)), recall = TP/(TP+false negatives (FN))). *Partial* precision and recall were calculated by defining a maximum cardinality minimum weight matching M between each computed transcript and the true transcripts as follows. Let $k \in 1 : K_C$ and $k \in 1 : K_T$ be the set of estimated and true isoforms respectively which are Boolean vectors of length E exons $\{1, 2, ..., E\}$, where a 1 at position e signifies that exon e is contained within that isoform and $k[e]$ indexes the position of the Boolean vector k. We define the distance between an inferred and true isoform d_{k_T,k_C} for all $k_T \in K_T$ and $k_C \in K_C$ to be the Hamming distance. The Hamming distance counts the number of mismatched exons between the estimated and true isoforms. The maximum cardinality minimum weight M is then the solution to the optimization problem

$$\min M = \sum_{k \in 1:K_C} \sum_{l \in 1:K_T} x_{kl} d_{kl} \tag{1}$$

$$\textbf{s.t.} \sum_{k \in 1:K_C} x_{kl} = 1 \qquad \forall l \in 1 : K_T \tag{2}$$

$$\sum_{l \in 1:K_T} x_{kl} = 1 \qquad \forall k \in 1 : K_C \tag{3}$$

$$x_{kl} \in \{0, 1\} \qquad \forall k \in 1 : K_C, \forall l \in 1 : K_T \tag{4}$$

For ease of computation and to ensure conditions 2 and 3 hold, the size of the true and inferred isoform sets must be balanced. Therefore, we add *dummy* isoforms to the smaller set which are connected to each isoform on the opposing set with a large distance $\gg \max(d_{ij})$. If the total number of isoforms is I, the augmented assignment problem can be solved in $O(I^3)$ time[15]. Let $M(k)$ be the d_{kl} in matching M for estimated isoform $k \in K_C$. If $M(k) = 0$, ($M(k) > 0$) then k is a true (false) positive; if $M(k) \leq p|E|$ ($M(k) > p|E|$) then k is a p-partial true (false) positive (p-TP and p-FP). Any true isoform not matched by a p-partial true positive is a p-partial false negative (p-FN). Using these definitions of p-TP, p-FP, and p-FN, we can compute p-precision and p-recall as above. Note that when $p = 0$, p-TP, p-FP, and p-FN reduce to true and false positives and false negatives.

For completeness, we also define an evaluation measure based on percentage of exons cov-

ered in the matched isoform solution. The *true exon coverage* is defined as the total number of bases of the true isoform covered by a matched isoform normalized by the true isoform length. *Inferred exon coverage* is defined as twice the total number of bases in the intersection between the inferred and true isoforms normalized by their total length. BIISQ obtains the highest scores for true and inferred exon coverage across different thresholds and isoform matching conditions (Supplementary Table 1).

Bayesian nonparametric discovery of isoforms and individual specific quantification (BIISQ)
In the following model specification, let there be M input samples (indexed by j) each with N_j reads (indexed by i). A particular gene contains E exons (indexed by e) and the set of global and individual specific isoforms are indexed by k and l respectively. Random variable subscripts indicated by an asterisk denote the full ordered set of indices. We assume RNA-seq reads have been aligned to the transcriptome and our observations are V read-terms which are compositions of the mapped position and sequence.

In our model, our observations are the reads from multiple samples, and our goal is to infer both the global and local sample-specific distributions of the isoforms. We represent each isoform as a probability vector over read terms, as inspired by the topic modeling application using hierarchical Dirichlet process (HDP)[16]. However, an important aspect of isoform discovery is inferring the exon composition of each isoform. We achieve the interpretability by explicitly modeling the isoforms and their exon compositions. We start from introducing the following latent variables. We denote the collection of global isoform as $\boldsymbol{\beta} = (\boldsymbol{\beta}_k)_{k=1}^{\infty}$, where each isoform $\boldsymbol{\beta}_k$ is sampled from a Dirichlet distribution regularized by the exons composition.

Let $\mathbf{b_k} = (\mathbf{b_{k1}}, \mathbf{b_{k2}}, \cdots \mathbf{b_{kV}})$ representing the read term composition of an isoform k. Recall that trivially, the V read terms are unique and we assume a consistent ordering of the read terms. Similarly, $\mathbf{b'_k} = (\mathbf{b'_{k1}}, \mathbf{b'_{k2}}, \cdots \mathbf{b'_{kE}})$ is a vector of discrete variable capturing whether exon e is included in the kth isoform ($b'_{ke} = 1$) or not ($b'_{ke} = 0$), which is modeled by a Bernoulli distribution with isoform-specific parameter π_k. We further sample π_k from a beta prior and thus this beta-Bernoulli hierarchy produces a beta posterior.

Generative model for BIISQ

The generative model of read terms $\{x_1, \dots, x_v, \dots, x_V\}$ for isoform k is modeled as a Dirichlet distribution β_k and represented as the normalized vector of V independent Gamma distributions.

$$\begin{aligned} \beta_k &\sim Dirichlet(b_{k1}\eta_1, \dots, b_{kV}\eta_V) \quad && \boldsymbol{\beta}_k \in \mathbb{R}^V \\ b_{kv} &\sim \text{Bernoulli}(\pi_\iota), && \text{for } \iota \in \{1, 2, \cdots, E\} \\ \pi_\iota &\sim \text{Beta}(r, s). \end{aligned} \tag{5}$$

where $b_{kv} \sim \text{Bernoulli}(\pi_\iota)$ for read term v that starts in exon ι.

Next, we denote the global proportion of an isoform β_k as t_k. BIISQ assumes a Dirichlet process prior on the global isoforms with the base distribution $U_{\mathbb{N}}$ and a concentration parameter ω,

$$G_0 \sim \text{DP}(\omega, U_{\mathbb{N}}). \tag{6}$$

Here $U_{\mathbb{N}}$ denotes the countably infinite set of positive integers, whose binary representation is used to denote the set of all possible isoform collections. Following the sticking-breaking process in[16], we can represent the global isoform distribution as

$$G_0 = \sum_{k=1}^{\infty} t_k \delta_{\boldsymbol{\beta}_k}, \tag{7}$$

where $\delta_{\boldsymbol{\beta}_k}$ represents the probability measure concentrated at isoform $\boldsymbol{\beta}_k$ and has the proportion given by the stick length t_k. The proportion for each isoform t_k is generated by the following distributions

$$\begin{aligned} t'_k &\sim \text{Beta}(1, \omega), \\ t_k &= t'_k \prod_{x=1}^{k-1} (1 - t'_x). \end{aligned} \tag{8}$$

To model sample-specific isoform distributions, we sample it from a Dirichlet process with base distribution as the global isoform distribution G_0 and concentration parameter α:

$$G_j | \alpha, G_0 \sim \text{DP}(\alpha, G_0). \tag{9}$$

Specifically, a sample-specific isoform distribution consists of a sample-specific collection of isoforms indexed by $\boldsymbol{c_j} = (c_{jl})_{l=1}^{\infty}$ and corresponding proportions $\boldsymbol{\psi_j} = (\psi_{jl})_{l=1}^{\infty}$. The indices of sample-specific isoforms c_{jl} is sampled from the global isoform distribution by

$$c_{jl} \sim \text{Multinomial}(\boldsymbol{t}), \boldsymbol{t} = (t_k)_{k=1}^{\infty}. \tag{10}$$

Following the stick-breaking process again, we can write the sample-specific isoform distribution as

$$G_j = \sum_{l=1}^{\infty} \psi_{jl} \delta_{c_{jl}}, \tag{11}$$

with the sample-specific isoform proportions

$$\begin{aligned} \psi'_{jl} &\sim \text{Beta}(1, \alpha), \\ \psi_{jl} &= \psi'_{jl} \prod_{x=1}^{l-1} (1 - \psi'_{jx}). \end{aligned} \tag{12}$$

Because draws from the base distribution are discrete, the individual isoform Dirichlet process priors will share global isoform atoms with probability 1. Moreover, the Dirichlet process as a prior distribution on partitions favors larger clusters; that is, the probability of a new observation joining an existing cluster is proportional to the size of that cluster, leading to a *rich-get-richer* phenomenon.

Finally, for each read we observed from the jth sample, denoted as w_{ji}, we first model the corresponding isoform assignment by z_{ji}, which is drawn from a multinomial distribution based on the sample-specific isoform proportion $\boldsymbol{\psi_j} = (\psi_{jl})_{l=1}^{\infty}$:

$$z_{ji} \sim \text{Multinomial}(\boldsymbol{\psi_j}). \tag{13}$$

Based on the assignment to the sample-specific isoforms, We model each read from a multinomial distribution with probability vector determined by the true global isoforms, or

$$w_{ji} \sim \text{Multinomial}(\boldsymbol{\beta}_{c_{jl'}}), \quad l' = z_{ji}. \tag{14}$$

Supplementary Figure 20 summarized the hierarchical generative model we described above. Once the model is fitted to mapped RNA-seq data, BIISQ parameters may be interpreted as biologically informative estimates of:

- $\boldsymbol{\beta}$: the isoforms in the population;
- t_k: the proportion of each isoform in the population;
- $\boldsymbol{\psi_j}$: the proportions of the isoforms in each sample j;
- $\boldsymbol{b'_k}$: exon composition or the probability of inclusion of each exon in each isoform.

Stochastic variational inference

We developed a stochastic variational inference (SVI) method to tractably and robustly estimate posterior probabilities in the BIISQ model, following prior work on SVI for the hierarchical Dirichlet process (HDP)[17]. We modified this method for the BIISQ-specific model parameters as follows. To handle the expansion and contraction of the population-wide isoforms, we implemented a split-merge step in SVIand executed this step every 30 iterations.

Variational inference combined with the mean-field assumption converts inference into a tractable optimization problem and one of the most frequently used techniques for optimization is coordinate ascent. In coordinate ascent, we iterate through variational parameters optimizing a single factor while keeping other variables fixed. It is guaranteed to converge to a local optimum. [17] derived the variational update equations for the similar HDP model, which are repeated here for completeness; BIISQ has additional model parameters so capture the composition of isoforms and these updates are derived below.

We define a variational parameter for each model variable in Supplementary Table 9.

If the maximum number of isoforms globally and individually is K and T respectively, then the truncated variational distribution is:

$$\begin{aligned} q(\beta, z, \psi, c, t, b, \pi) &= \left(\prod_{k=1}^{K} q(t_k|a_k^1, a_k^2) q(\pi_k|\rho_k) q(\beta_k|\lambda_k) \prod_{e=1}^{E} q(b'_{ke}|\mu_{ke})\right) \\ &\quad * \left(\prod_{j=1}^{M}\prod_{l=1}^{T} q(\psi_{jl}|\gamma_{jl}^1, \gamma_{jl}^2) q(c_{jl}|\zeta_{jl}) \prod_{j=1}^{M}\right) * \left(\prod_{i=1}^{N_j} q(z_{ij}|\phi_{ij})\right) \end{aligned}$$

The complete conditional distributions for variables z, ψ, c, t remain unchanged with respect to[17], however, the β_k is modeled with V gamma distributed random variables $(\tilde{\beta_{kv}})_{v=1}^{V}$.

$$\tilde{\beta_{kv}}|z, c, w, b', exons, \eta \ \sim \ Dirichlet\left(\mathbf{b}_k \cdot \left(\eta + \sum_{j=1}^{M}\sum_{l=1}^{T}\mathbb{I}_{c_{jl}=k}\sum_{i=1}^{N_j}\mathbb{I}_{z_{ji}=c_{jl}}\mathbb{I}_{w_{ji}=v}\right)\right) \tag{15}$$

The components of the Dirichlet are only valid if the read-term v maps to exon b'_k. The inner sum counts the number of reads mapping to individual isoform l while the outer double sum accounts for multiple individuals and the mapping between individual isoform and global isoform.

The exon-mapping **b'** and read-term mapping **b** variables are novel to BIISQ; we marginalize over the π variables, but **b'** is an interpretable indicator for isoform composition and thus derive its variational update below. Let S be the set of read-terms $(x_v)_{v=1}^{|S|}$ containing the exon $e \in \{min(M_v), ..., max(M_v)\}$. The conditional for b'_e is:

$$P(b'_{ke}|b_k, \tilde{\beta_k}, \pi, \beta, \kappa, \eta, exons) \propto \prod_{v \in S} P(\tilde{\beta_{kv}}|b'_k, b_k, \kappa, \eta) \int_{\pi} P(b'_{ke}|\pi, exons) P(\pi|r, s) \tag{16}$$

which is a product of Gamma distributions and a beta-Bernoulli. Unfortunately, this is not a known distribution and we cannot exploit conjugacy. We derive the variational update $q_e^*(b'_e)$ using the

expected value of the exponentiated log of the conditional distribution.

$$
\begin{aligned}
q^*_{ke}(b'_{ke}) &\propto exp\left\{\mathbb{E}_{-b'_{ke}}\left[\log\prod_{v\in S}P(\tilde{\beta_{kv}}|\mathbf{b}'_k,\mathbf{b}_k,\eta,\kappa)+\log\int_\pi P(b'_{ke}|\pi,exons)P(\pi|r,s)\right]\right\} && (17)\\
&= exp\left\{\mathbb{E}_{-b'_{ke}}\left[\log\prod_{v\in S}Gamma(b_{kv}\eta,\kappa)+\log\int_\pi Bern(b'_{ke}|\pi)Beta(\pi|r,s)\right]\right\} && (18)\\
&= exp\Bigg\{\mathbb{E}_{-b'_{ke}}\left[\log\prod_{v\in S}\left(\frac{1}{\Gamma(b_k\eta)\kappa^{b_k\eta}}\tilde{\beta_{kv}}^{b_k\eta-1}e^{-\tilde{\beta_{kv}}/\kappa}\right)\right] && (19)\\
&\quad+\mathbb{E}_{-b'_{ke}}\left[\log\left(\frac{(b'_e)^{r-1+\mathbb{I}_{b'_e=1}}(1-b'_e)^{s-1+\mathbb{I}_{b'_e=0}}}{B(r,s)}\right)\right]\Bigg\} && (20)\\
&= exp\Bigg\{\mathbb{E}_{-b'_{ke}}\left[\sum_{v\in S}-\log\Gamma\left(b_k\eta\right)-b_k\eta\log\left(\kappa\right)+\left(b_k\eta-1\right)\log\tilde{\beta_{kv}}-\tilde{\beta_{kv}}/\kappa\right] && (21)\\
&\quad+(r-1+\mathbb{I}_{b'_e=1})\log\left(b'_e\right)+\left(s-1+\mathbb{I}_{b'_e=0}\right)\log\left(1-b'_e\right)\Bigg\} && (22)\\
&= exp\Bigg\{\sum_{v\in S}-\mathbb{E}_{-b'_{ke}}\left[\log\Gamma\left(b_k\eta\right)\right]-\mathbb{E}_{-b'_{ke}}[b_k]\eta\log\left(\kappa\right) && (23)\\
&\quad+\mathbb{E}_{-b'_{ke}}\left[\left(b_k\eta-1\right)\log\tilde{\beta_{kv}}\right]-\frac{1}{\kappa}\mathbb{E}_{-b'_{ke}}[\tilde{\beta_{kv}}] && (24)\\
&\quad+(r-1+\mathbb{I}_{b'_e=1})\log\left(b'_e\right)+\left(s-1+\mathbb{I}_{b'_e=0}\right)\log\left(1-b'_e\right)\Bigg\} && (25)\\
& && (26)
\end{aligned}
$$

$$
\begin{aligned}
q_e^*(b_e') &\propto exp\left\{\mathbb{E}_{-e}\left[\log Dir(\beta|b_e', b_{-e}', \eta, exons) + \log \int_\pi Bern(b_e'|\pi)Beta(\pi|r,s)\right]\right\} \\
&\propto exp\Bigg\{\mathbb{E}_{-e}\left[\log\left(\frac{\Gamma(\sum_{v=1}^V \eta * b_{kv})}{\prod_{v=1}^V \Gamma(\eta * b_{kv})}\prod_{v=1}^V \beta_v^{(\eta * b_{kv}-1)}\right)\right] + \\
&\quad \mathbb{E}_{-e}\left[\log\left(\frac{(b_e')^{r-1+\mathbb{I}_{b_e'=1}}(1-b_e')^{s-1+\mathbb{I}_{b_e'=0}}}{B(r,s)}\right)\right]\Bigg\} \\
&\propto exp\Bigg\{\mathbb{E}_{-e}\left[\log\left(\Gamma\left(\sum_{v=1}^V \eta * b_{kv}\right)\right) - \log\left(\prod_{v=1}^V \Gamma(\eta * b_{kv})\right) + \log\left(\prod_{v=1}^V \beta_v^{(\eta * b_{kv}-1)}\right)\right] \\
&\quad +(r-1+\mathbb{I}_{b_e'=1})\log(b_e') + (s-1+\mathbb{I}_{b_e'=0})\log(1-b_e')\Bigg\} \\
&\sim exp\Bigg\{\log\left(\Gamma\left(\sum_{v=1}^V \eta * b_{kv}\right)\right) - \sum_{v=1}^V \log(\Gamma(\eta * b_{kv})) + \sum_{v=1}^V (\eta * b_{kv} - 1)\mathbb{E}_{-e}[\log(\beta_v)] \\
&\quad +(r-1+\mathbb{I}_{b_e'=1})\log(b_e') + (s-1+\mathbb{I}_{b_e'=0})\log(1-b_e')\Bigg\}
\end{aligned}
$$

where $\mathbb{I}_{b_e'=x}$ is an indicator function for the exon corresponding to b_e' being in isoform k and b_{kv} indicates if read term v maps to isoform k. An exon is either included in the isoform or not, we compute the update for $q_e^*(b_e')$ with $e = 0$ and also with $e = 1$ and then normalize.

Additional notes for the μ update

To update the variational parameter for β_k we iterate through each exon and compute the probability conditional on the exon being turned on and off. This involves computing the expectation of a log Gamma function of a sum of b_{kv}. We approximate this expectation using the variational parameter μ in the summation; tests comparing monte carlo estimates to this approximation showed that the correlation is very high.

Initialization The total number of global and sample specific isoforms can be as large as the data support, but, we truncate the total number of possible isoforms to $2^E - 1$, the theoretical

maximum. For genes with many exons, $2^E - 1$ is prohibitively large and initializing the exon selector $\boldsymbol{b'_k}$ vector with $2^E - 1$ isoforms is computationally intractable. There are several different methods for initializing isoforms.

1. random: generate a random binary vectors corresponding to random isoforms
2. random: generate isoforms with exon inclusion probabilities following the Beta prior distribution
3. read-term naive: while there is a read-term without an alignment to an isoform, generate a random isoform with the exons specified in read-term
4. read-term cluster: cluster read-terms that are consistent. Generate an isoform for each cluster.

In this work, we simply initialize a single isoform that includes each exon.

Read mapping likelihood

Let the partial read counts for an isoform k be

$$(\tilde{n_k})_{v=1}^{V} = \left(\sum_{j=1}^{M} \sum_{l=1}^{T} \zeta_{jl}^{k} \sum_{i=1}^{N_j} \phi_{ji}^{l} * \mathbb{I}_{w_{ji}=v} \right) \tag{27}$$

Then, the likelihood for reads w_{ji} for isoform k is

$$P(w_{ji}|\zeta_{ji}, \phi_{ji}, \lambda_k) = \prod_{\forall j} \frac{\Gamma\left(\left(\sum_{v=1}^{V} n_k^v\right) + 1\right) \Gamma\left(\sum_k \lambda_{kv}\right)}{\Gamma\left(\sum_{v=1}^{V} n_k^v + \sum_v \lambda_{kv}\right)} \prod_{v=1}^{V} \frac{\Gamma\left(n_k^v + \lambda_{kv}\right)}{\Gamma\left(n_k^v + 1\right) \Gamma\left(\lambda_{kv}\right)}$$

Details on read term computation.

BIISQ represents transcripts internally as sequences of contiguous transcribed regions. A *read term* is the principle data structure which describes how the input aligned read fragments interact with

Algorithm 1: Stochastic variational inference BIISQ.

input : V read-terms, N_j reads for j individuals, convergence criteria

init : isoforms μ, Dirichlet parameters λ using μ and b_{kv}, $a_1 = 1, a_2 = \omega$, step size δ_t, SVI forgetting rate=0.7, SVI delay=1.0

output: $\mu_{ke}, \gamma_{jl}, \phi_{ij}$

while *(likelihood not converged)* **do**
- // Update individual plate
- **while** *(local parameters not converged)* **do**
  - **for** j *in* $(1 : M)$ **do**
    - **for** l *in* $(1 : T)$ **do**
      - $\gamma_{jl}^1 \leftarrow 1 + \sum_{i=1}^{N_j} \phi_{ji}^l$
      - $\gamma_{jl}^2 \leftarrow \alpha + \sum_{i=1}^{N_j} \sum_{x>l} \phi_{ji}^x$
      - **for** k *in* $(1 : K)$ **do**
        - $\zeta_{jl}^k \propto \exp\left(E_q \log t_k + \sum_{i=1}^{N_j} E_q \mathbb{I}(z_{ji} = c_{jl}) E_q \log \beta_{k,w_{ji}}\right)$
      - **for** i *in* $(1 : N_j)$ **do**
        - $\phi_{ji}^l \propto \exp(E_q \log \psi_{jl} + \sum_{k=1}^{K} E_q \mathbb{I}(c_{jl} = k) E_q \log \beta_{k,w_{ji}})$
- // Update isoform plate
- **for** k *in* $(1 : K)$ **do**
  - $\hat{a}_k^1 \leftarrow 1 + \sum_{j=1}^{M} \sum_{l=1}^{T} \zeta_{jl}^k$
  - $\hat{a}_k^2 \leftarrow \omega + \sum_{j=1}^{M} \sum_{l=1}^{T} \sum_{x>k} \zeta_{jl}^x$
  - **for** v *in* $(1 : V)$ **do**
    - $\hat{\lambda}_k^v \leftarrow E_q b_{kv} * \left(\eta + \sum_{j=1}^{M} \sum_{l=1}^{T} \zeta j l^k \sum_{i=1}^{N_j} \phi_{ji}^l * \mathbb{I}_{w_{ji}=v}\right)$
  - **for** e *in* $(1 : E)$ **do**
    - $\hat{\mu}_{ke} \leftarrow\propto exp\left\{\mathbb{E}_{-e}\left[\log Dir(\beta | b'_e, b'_{-e}, \eta, exons) + \log \int_\pi Bern(b'_e | \pi) Beta(\pi | r, s)\right]\right\}$
- $a^t \leftarrow (1 - \delta_t) a^{t-1} + \delta_t \hat{a}$
- $\lambda^t \leftarrow (1 - \delta_t) \lambda^{t-1} + \delta_t \hat{\lambda}$
- $\mu^t \leftarrow (1 - \delta_t) \mu^{t-1} + \delta_t \hat{\mu}$

transcripts (see Online Methods); it is a tuple of a mapped read's terminal basepair positions and the set of covered exons. The set of read terms for all individuals and all references are computed in two passes. The first pass converts sequence read alignments (BAM or BED file) and a set of gene references to a file where each line denotes the read term (terminal bases and covered exons) and the aligned reference. This process is repeated for each sample. The second pass aggregates all read terms across samples for a single reference produces the input to BIISQ: a file describing the counts of the read terms across samples and a file defining each read term.

Supplementary References

1. Data Release: Whole Human Transcriptome from Brain, Heart, and Liver. `http://www.pacb.com/blog/data-release-whole-human-transcriptome/` (2016).

2. ENCODE Project Consortium *et al.* An integrated encyclopedia of DNA elements in the human genome. *Nature* **489**, 57–74 (2012).

3. Ernst, J. & Kellis, M. Discovery and characterization of chromatin states for systematic annotation of the human genome. *Nature biotechnology* **28**, 817–825 (2010).

4. Grant, G. R. *et al.* Comparative analysis of RNA-Seq alignment algorithms and the RNA-Seq unified mapper (RUM). *Bioinformatics* **27**, 2518–2528 (2011).

5. Bioinfx study: Optimizing STAR aligner for Iso Seq data. `https://github.com/PacificBiosciences/cDNA_primer/wiki/` (2015).

6. Lappalainen, T. *et al.* Transcriptome and genome sequencing uncovers functional variation in humans. *Nature* **501**, 506–511 (2013).

7. Trapnell, C. *et al.* Transcript assembly and quantification by RNA-Seq reveals unannotated transcripts and isoform switching during cell differentiation. *Nature Biotechnology* **28**, 511–515 (2010).

8. Li, W., Feng, J. & Jiang, T. IsoLasso: A LASSO Regression Approach to RNA-Seq Based Transcriptome Assembly. *Journal of Computational Biology* **18**, 1693–1707 (2011).

9. Li, J. J., Jiang, C.-R., Brown, J. B., Huang, H. & Bickel, P. J. Sparse linear modeling of next-generation mRNA sequencing (RNA-Seq) data for isoform discovery and abundance estimation. *Proceedings of the National Academy of Sciences* **108**, 19867–19872 (2011).

10. Tasnim, M., Ma, S., Yang, E.-W., Jiang, T. & Li, W. Accurate inference of isoforms from multiple sample RNA-Seq data. *BMC Genomics* `https://doi.org/10.1186/1471-2164-16-S2-S15` (2015).

11. Wu, T. D. & Watanabe, C. K. GMAP: a genomic mapping and alignment program for mRNA and EST sequences. *Bioinformatics* **21**, 1859–1875 (2005).

12. Dobin, A. *et al.* STAR: ultrafast universal RNA-seq aligner. *Bioinformatics* **29**, 15–21 (2013).

13. Li, W. & Jiang, T. Transcriptome assembly and isoform expression level estimation from biased RNA-Seq reads. *Bioinformatics* **28**, 2914–2921 (2012).

14. Steijger, T. *et al.* Assessment of transcript reconstruction methods for RNA-seq. *Nature Methods* **10**, 1177–1184 (2013).

15. Edmonds, J. & Karp, R. M. Theoretical improvements in algorithmic efficiency for network flow problems. *J. ACM* **19**, 248–264 (1972).

16. Teh, Y. W., Jordan, M. I., Beal, M. J. & Blei, D. M. Hierarchical Dirichlet Processes. *Journal of the American Statistical Association* **101**, 1566–1581 (2006).

17. Hoffman, M. D., Blei, D. M., Wang, C. & Paisley, J. W. Stochastic variational inference. *Journal of Machine Learning Research* **14**, 1303–1347 (2013).
