# Supplementary material for: Bayesian nonparametric discovery of isoforms and individual specific quantification
Source: Nat Commun. 2018 Apr 27;9:1681. doi: 10.1038/s41467-018-03402-w (PMC5923247; doi:10.1038/s41467-018-03402-w)
Supplement: Supplementary file 3 — Description of Additional Supplementary Files [file 41467_2018_3402_MOESM3_ESM.pdf]

Description of Additional Supplementary File

File Name: Supplementary Data 1
Description: **Transcript ratio QTLs and population and sex-specific transcripts in the GEUVADIS data.** The first two tables show transcript IDs and p-values for the 924 and 148 significant ($\chi 2$ test, Bonferroni-corrected $p \leq 0.05$) population-and sex-specific transcript ratio distributions. The last table displays transcript ratio QTLs identified by Matrix eQTL with $p \leq 0.01$.
